# Site-directed biochemical analyses reveal that the switchable C-terminus of Rpc31 contributes to RNA polymerase III transcription initiation

**DOI:** 10.1093/nar/gkac1163

**Published:** 2022-12-09

**Authors:** Arvind Chandra Shekhar, Yuan-En Sun, Seok-Kooi Khoo, Yu-Chun Lin, Ester Betaria Malau, Wei-Hau Chang, Hung-Ta Chen

**Affiliations:** Institute of Molecular Biology, Academia Sinica, Taipei, Taiwan, R.O.C; Institute of Molecular Biology, Academia Sinica, Taipei, Taiwan, R.O.C; Institute of Molecular Biology, Academia Sinica, Taipei, Taiwan, R.O.C; Institute of Molecular Biology, Academia Sinica, Taipei, Taiwan, R.O.C; Institute of Molecular Biology, Academia Sinica, Taipei, Taiwan, R.O.C; Institute of Chemistry, Academia Sinica, Taiwan, R.O.C; Institute of Molecular Biology, Academia Sinica, Taipei, Taiwan, R.O.C

## Abstract

Rpc31 is a subunit in the TFIIE-related Rpc82/34/31 heterotrimeric subcomplex of *Saccharomyces cerevisiae* RNA polymerase III (pol III). Structural analyses of pol III have indicated that the N-terminal region of Rpc31 anchors on Rpc82 and further interacts with the polymerase core and stalk subcomplex. However, structural and functional information for the C-terminal region of Rpc31 is sparse. We conducted a mutational analysis on Rpc31, which uncovered a functional peptide adjacent to the highly conserved Asp-Glu-rich acidic C-terminus. This C-terminal peptide region, termed ‘pre-acidic’, is important for optimal cell growth, tRNA synthesis, and stable association of Rpc31 in the pre-initiation complex (PIC). Our site-directed photo-cross-linking to map protein interactions within the PIC reveal that this pre-acidic region specifically targets Rpc34 during transcription initiation, but also interacts with the DNA entry surface in free pol III. Thus, we have uncovered a switchable Rpc31 C-terminal region that functions in an initiation-specific protein interaction for pol III transcription.

## INTRODUCTION

Eukaryotic RNA polymerase III (pol III) is responsible for transcribing tRNAs, 5S ribosomal RNA, U6 spliceosomal RNAs, small nucleolar RNAs, and microRNAs (1). The pol III transcription machinery is highly conserved among eukaryotes (2–4). In *Saccharomyces cerevisiae* (yeast), the pol III complex consists of a total of 17 subunits, and its catalytic core comprises 12 subunits (5–8). The remaining five subunits form two pol III-specific subcomplexes: the Rpc82/34/31 trimer and the Rpc53/37 dimer. A previous analysis comparing the pol II and pol III machineries indicated that the pol III-specific subunits are related to the pol II basal transcription factors TFIIE and TFIIF that are permanently bound to the polymerase core (9). Structural analyses have also indicated that all TFIIE- and TFIIF-related complexes mainly contact the molecular surface of the active site cleft to associate with the polymerase core (4). In pol III, the active site cleft is formed by the two largest subunits, i.e. Rpc160 and Rpc128.

**Figure 1.**
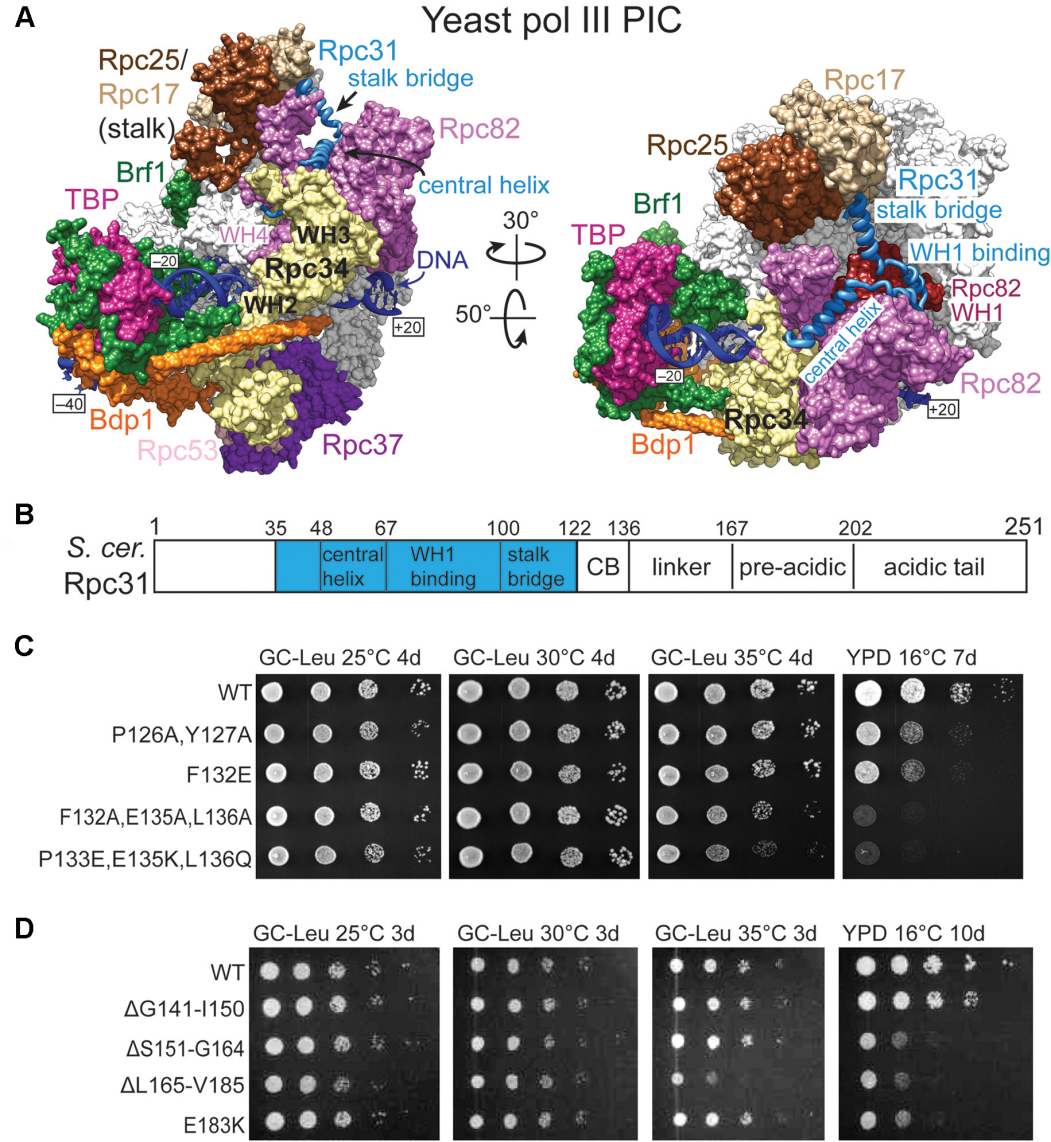
Pol III PIC model and genetic analysis of Rpc31. (**A**) Two orientations of a pol III PIC model consisting of pol III, TFIIIB and DNA (PDB code: 6eu0). The 10-subunit pol III core is colored grey, and the template and non-template DNA strands are in dark blue. The Rpc25/17, Rpc53/37 and Rpc82/34/31 subcomplexes are colored as indicated. The Rpc31 subunit is shown as a blue backbone trace model. The WH1 domain of Rpc82 is colored red. The Rpc34 subunit is colored yellow. WH: winged-helix domain. TFIIIB subunits: Brf1 (green), TBP (magenta), and Bdp1 (orange). (**B**) Structural domains in Rpc31. Unstructured regions are in white. The structural illustration was generated by the Chimera program (34). (**C**) Cell growth analysis (by serial-dilution spot assay) of Rpc31 mutant lines harboring mutations in the conserved block (CB) region (aa 122–136). GC-leu, synthetic complete + glucose medium without leucine. YPD, yeast extract + peptone + dextrose medium. d, days. (**D**) Cell growth analysis of Rpc31 mutant lines harboring mutations in the linker and pre-acidic regions (aa 136–202).

The Rpc82/34/31 trimer is regarded as a TFIIE-related subcomplex based on the multiple winged-helix (WH) domains of Rpc82 and Rpc34 being similar to those of the two TFIIE subunits in the pol II machinery (4,9). Cryo-electron microscopy (cryo-EM)-based structural analyses on the initiating pol II and pol III complexes have demonstrated that the WHs of Rpc82/34 and TFIIE are essential structural elements for the protein–DNA network on the polymerase active site cleft (6,7,10). For example, the Rpc82/34/31 subcomplex contacts the Rpc160 clamp domain through the Rpc82-WH1/WH4 and Rpc34-WH3 domains, whereas Rpc34-WH2 and the Rpc82-WH4 cleft loop interact with the DNA bubble (Figure 1A). In support of their structural positions, Rpc34 and Rpc82 have been characterized as functioning in DNA opening and stabilization of the pre-initiation complex (PIC) (11–13). The third subunit, Rpc31–a 251-amino acid protein, contains stretches of Asp and Glu residues ranging from residues 202 to 248 of the C-terminus (Figure 1B) (14). Genetic and biochemical studies have demonstrated that this Asp-Glu C-terminus, also referred to as the acidic tail, is important for cell viability and transcription initiation of pol III-specific genes, but does not affect elongation, termination and recycling of pol III (14,15).

Yeast Rpc31 has no paralogous counterpart in the pol I and II enzymes (15). Rpc31 is highly homologous to the Rpc7α/β (Rpc32α/β) isoforms in human and higher eukaryotes (16). Rpc7β is ubiquitously expressed in all tissues and it is essential for cell growth, whereas Rpc7α expression is restricted to undifferentiated embryonic stem cells and tumor cells (16). The co-crystal structure of Rpc7β and Rpc3 (also referred to as Rpc62; a homolog of yeast Rpc82) indicates that the N-terminal region of Rpc7 forms an extended structure to bind with the first two WHs of Rpc3 (17). This Rpc3/Rpc7 binding mode was further evidenced by subsequent structural analyses of the human pol III complex (18,19). In the resulting cryo-EM structures of elongating human pol III complex, the N-terminal region of Rpc7α sequentially folds into a polymerase core binding loop, a Rpc3-binding central helix, and a stalk bridge loop (Supplementary Figure S1A and B). In addition, in the structure of unbound human pol III, the Asp-Glu acidic tail of Rpc7 was partially resolved as localizing in the polymerase active site tunnel, suggesting it functions to inhibit binding of non-promoter DNA to pol III (Supplementary Figures S1A and C) (19). Structural analyses of the yeast pol III complex have also indicated that Rpc31 possesses a structural arrangement similar to its human counterpart (Rpc7) (6). In the cryo-EM structure from that study of an initiating yeast pol III complex with an open DNA and the transcription factor TFIIIB, a ∼90-residue peptide sequence in the N-terminal region of Rpc31 folds into a central helix and a stalk bridge that binds respectively to Rpc82 and the Rpc25/17 stalk dimer (Figures 1A and B). The entire C-terminal peptide sequence following the stalk bridge helix is unstructured. Based on sequence homology with human Rpc7, the Asp-Glu acidic tail of yeast Rpc31 could also reside in the active site tunnel in the absence of DNA (19).

Thus, recent advances in structural analyses of yeast pol III have provided structural data for the N-terminal region of Rpc31. However, structural data for its C-terminal region are lacking and, apart from the Asp-Glu acidic tail, the functions of this peptide region had remained unclear. We conducted a mutational analysis on yeast Rpc31, revealing a requirement for the unstructured pre-acidic region proximal to the acidic tail for optimal cell growth. This pre-acidic region functions in pol III transcription by supporting the stable association of Rpc31 in the PIC. We conducted a photo-cross-linking analysis to map the protein interactions of Rpc31 within the PIC, revealing interaction sites for Rpc34 in the pre-acidic region. Further protein binding analysis using microscale thermophoresis (MST) confirmed a requirement for this pre-acidic region in Rpc34 interaction. Thus, our genetic, biochemical, and biophysical analyses have revealed an unappreciated C-terminal region of Rpc31 that supports the protein network responsible for pol III transcription initiation.

## MATERIALS AND METHODS

### Yeast strains and plasmids

For our *Rpc31* mutagenesis study, we employed a yeast (*Saccharomyces cerevisiae*) strain for plasmid shuffling derived from BY4705 (20). The chromosomal *Rpc31* gene in BY4705 was disrupted with the KanMX4 cassette, yielding strain YC31 (*MAT*α *ade2::his3G his3*Δ*200 leu2*Δ*0 met15*Δ*0 lys2*Δ*0 trp1*Δ*63 ura3*Δ*0* [*RPC31::KanMX4*] *RPC31*-pRS316 (*URA3^+^*)). The gene encoding *Rpc31*, along with the endogenous promoter and a single V5-epitope tag at its C-terminus, was cloned into the pRS315 vector, yielding the pC31 (*RPC31-*V5, *ars cen, LEU2*) plasmid. All Rpc31 mutant plasmids were derived from pC31 by *in vitro* mutagenesis. Rpc31 mutant strains were separately generated by growing transformed strains on 5-FOA-containing medium to replace the *URA3*-hosting *RPC31*-pRS316 plasmid with *LEU2*-hosting mutant plasmids. For cell growth assays, strains harboring wild-type (WT) or mutant Rpc31 were grown in YPD to an optical density (600 nm) of 1.0, and the cell cultures were subsequently diluted from 10^−1^ to 10^−4^. The diluted cells were spotted onto synthetic complete glucose plates lacking leucine, and their growth phenotypes were monitored at 16, 25, 30 and 37°C. The incubation time for cell growth at 30 and 37°C was 3 days, and at 16°C it was 7 days.

We employed a plasmid shuffling strategy to incorporate the non-natural amino acid *p*-benzoyl-l-phenylalanine (BPA; Bachem) into Rpc31 and Rpc160. Genes encoding *Rpc31* and *Rpc160* were separately cloned into the yeast 2-micron vector pRS425 with the *LEU2* selection marker (21). Both genes were driven by the yeast *ADH1* promoter. The C-termini of *Rpc31* and *Rpc160* possessed a V5-epitope and 13 copies of the Myc-epitope, respectively. Hereafter, the constructed plasmids are referred to as pCJ1 (Adh1-*Rpc31* C-ter V5-pRS425) and pCJ2 (Adh1*-Rpc160* C-ter 13Myc-pRS425), respectively. To generate individual plasmids for subsequent BPA incorporation, the ‘TAG’ (amber) nonsense codon was introduced into the Rpc31 and Rpc160 coding sequences at intended amino acid positions by *in vitro* mutagenesis. The resulting plasmids, denoted ‘amber plasmids’, were transfected into respective shuffle strains to generate the mutant strains. To allow site-specific BPA substitutions in proteins through non-sense suppression, we co-transfected plasmid pLH157 containing coding sequences of the suppressor tRNACUA and BPA-tRNA synthetase (22). The yeast strain for Rpc160 plasmid shuffling is CPy1 (*MAT*α *ade2::his3G his3*Δ*200 leu2*Δ*0 met15*Δ*0 lys2*Δ*0 trp1*Δ*63 ura3*Δ*0* [*RPC160::KanMX4*] *RPC160*-pRS316 (*URA3^+^*)), which was generated in a previous study (12).

### Immunoprecipitation (IP)

To prepare yeast whole cell extract (WCE) for IP assays, 1-liter yeast cell culture was grown in YPD medium to an O.D. of 1.5. The harvested cells were lysed and processed for WCE preparation according to a protocol described previously (23,24). WCE (1.5 mg) was mixed with 75 μl of anti-FLAG antibody agarose beads (M2; Sigma-Aldrich) and incubated at 4°C for 2 h in WCE buffer (20 mM HEPES (pH 7.9), 100 mM KCl, 5 mM MgCl_2_, 1 mM EDTA and 20% glycerol). The bound proteins were washed three times with 200 μl of WCE buffer, and the proteins were extracted by boiling with NuPAGE sample buffer (Invitrogen) for subsequent SDS-PAGE and Western blot analyses. For all Western blot analyses in this study, immuno-stained protein bands were visualized using the Odyssey infrared imaging system (LI-COR Biosciences).

### *In vitro* transcription assay

*In vitro* transcription assays were conducted according to a protocol described previously using circular DNA plasmid and yeast WCE (23,24). In brief, WCE (50 μg) was pre-incubated with 150 ng plasmid DNA containing the *SUP4* (tRNA-tyr) gene in a final volume of 17 μl transcription buffer (20 mM HEPES (pH 7.9), 80 mM KCl, 5 mM MgCl_2_, 1 mM EDTA, 2% glycerol and 0.01% Tween 20), together with 200 ng α-amanitin, 4 units of RNase inhibitor (Roche), and 1 mM dithiothreitol (DTT). Transcription was started upon further addition of 3 μl NTPs [ATP (500 μM), UTP (500 μM), CTP (500 μM), GTP (50 μM) and [α-^32^P] GTP (0.08 μM [3000 Ci/mmol])] to a final volume of 20 μl. After reaction at 30°C for 30 min, RNA products were extracted and analyzed by denaturing polyacrylamide (6%) gel electrophoresis. The SUP4 pre-tRNA transcripts were visualized by autoradiography and quantified in ImageJ software (NIH Image).

### PIC isolation by immobilized template assay

To isolate the pol III PIC by means of immobilized template (IMT) assay, yeast WCEs from 1-liter cell culture were incubated with a 5′-end biotin-conjugated DNA fragment containing the *S. cerevisiae* SUP4 tRNA gene sequence, as detailed previously (23). Briefly, the 603-bp biotinylated DNA was amplified by polymerase chain reaction (PCR) and subsequently immobilized on Streptavidin magnetic beads (Dynal) in a transcription buffer containing 20 mM HEPES (pH 7.9), 80 mM KCl, 5 mM MgCl_2_, 1 mM EDTA, 2% glycerol, 0.01% Tween 20 and 1 mM DTT. Each WCE (800 μg) was mixed with 2 μg of immobilized DNA in a final volume of 100 μl transcription buffer for 30-min incubation at 30°C. The isolated PICs were washed three times with transcription buffer before undergoing Western blot analysis.

### BPA photo-crosslinking

Yeast cultures were grown in YPD medium containing BPA (Bachem). WCE preparation procedures for BPA cross-linking analysis using isolated PICs immobilized on a biotinylated DNA fragment containing the *SUP4* tRNA gene were as described in a previous publication (23). In brief, for a typical BPA photo-crosslinking experiment, 800 μg WCE was incubated with 4 μg DNA template immobilized on 20 μl Streptavidin magnetic beads (Dynal). After washing three times with transcription buffer (20 mM HEPES pH 7.9, 80 mM KCl, 5 mM MgCl_2_, 1 mM EDTA and 2% glycerol) containing 0.01% Tween 20, the reaction mix was divided into two fractions: one for UV-irradiation (+UV) and the other as a control (−UV). UV irradiation was conducted using a Spectrolinker XL-1500 UV oven (Spectronics) with a total energy of 7500 μJ cm^−2^. The isolated PICs were then resuspended in NuPAGE loading buffer (Invitrogen) for SDS-PAGE and Western blot analyses.

### Purification of Rpc34

The gene sequence for full-length Rpc34 protein with an N-terminal V5 epitope tag was cloned using the SpeI and SalI sites in the multiple cloning site of a pET21aHis_6_SUMO vector (Novagen), resulting in pET21aHisSUMOV5C34 plasmid. The confirmed pET21aHisSUMOV5C34 plasmid was transformed into *Escherichia coli* strain BL21 (DE3) RIL (Stratagene) for protein overexpression. Overexpression of Rpc34 was induced by addition of 0.4 mM IPTG and overnight growth at 16°C. Cells from 4 L of *E. coli* culture were harvested, lysed and purified using Ni-Sepharose (GE Healthcare) in lysis buffer (50 mM Tris–Cl (pH7.5), 500 mM NaCl, 5% glycerol, 20 mM imidazole). After lysis using a microfluidizer (Microfluidics), the cell extract was clarified by centrifugation and then subjected to Ni-Sepharose-based affinity purification. SUMO fusion polypeptides were eluted in elution buffer (50 mM Tris–Cl (pH7.5), 150 mM NaCl, 5% glycerol, 250 mM imidazole), and the eluates were subsequently dialyzed against buffer A (50 mM Tris–Cl (pH7.5), 150 mM KCl, 5% glycerol). All buffers were supplemented with 2 mM β-mercaptoethanol and phenylmethylsulphonyl fluoride (PMSF). The SUMO tag was subsequently removed by adding purified yeast Ulp1 (SUMO protease) to the eluate at a concentration of 1.76 μg/ml for 2 h at room temperature (RT). SUMO protease, SUMO tags and uncleaved fusion proteins were removed by means of a second passage through Ni-Sepharose. Buffer exchange was carried out on SUMO-digested Rpc34 using a NAP^TM^ 5 Column containing 10 mM Sodium Phosphate (pH 8.0) and 150 mM KCl. Eluted proteins were concentrated and stored at -80°C for further use.

### Purification of Rpc31 C-terminal peptides

The gene sequence for the C-terminal Rpc31 aa. 121–251 peptide with a C-terminal Flag epitope was cloned using the SacI and XhoI sites into the multiple cloning site of a pET21aHis_6_SUMO vector (Novagen), resulting in pET21aHisSUMOC31(121–251)-Flag plasmid. The confirmed pET21aHisSUMOC31(121–251)-Flag plasmid was transformed into *E. coli* strain BL21 (DE3) RIL (Stratagene) for protein overexpression. Rpc31(121–251)-Flag was induced by addition of 0.2 mM IPTG and growth at 30°C for 6 h. Cells from 4 L of *E. coli* culture were harvested, lysed and purified using Ni-Sepharose (GE Healthcare) using the lysis buffer (50 mM Tris–Cl (pH7.5), 500 mM NaCl, 5% glycerol, 20 mM imidazole). After lysis using a microfluidizer (Microfluidics), the cell extract was clarified by centrifugation and then subjected to Ni-Sepharose-based affinity purification. SUMO fusion polypeptides were eluted in elution buffer (50 mM Tris–Cl (pH7.5), 300 mM NaCl, 5% glycerol, 250 mM imidazole), and the eluates were subsequently dialyzed against buffer A (50 mM Tris–Cl (pH7.5), 150 mM KCl, 5% glycerol). All buffers were supplemented with 2 mM β-mercaptoethanol and PMSF. SUMO tags were subsequently removed by adding purified yeast Ulp1 (SUMO protease) to the eluate at a concentration of 1.76 μg/ml for 2 h at RT. SUMO protease, SUMO tags and uncleaved fusion proteins were removed by means of a second passage through Ni-Sepharose. The Rpc31 C-terminal peptides were further purified using a Source15Q column with a 10-column volume linear gradient from 160 mM to 1 M KCl. The Rpc31 C-terminal peptides eluted at ∼550 mM KCl. Eluted proteins were concentrated and stored at −80°C for further use.

### Microscale thermophoresis

Purified Rpc34 protein was labeled by applying a concentration of 20 μM protein to Alexa Fluor™ 647 NHS Ester (Thermo Fischer) (molar ratio of dye/protein = 3) at room temperature for 30 min in the dark. Unreacted dye in the labeling reaction was removed by passing the mixture through a PD Midi Trap™ G-25 (GE) desalting column, and the labeled protein was eluted using 500 μl of 5 mM sodium phosphate buffer. Labeling efficiency was determined as ∼0.8 according to UV/VIS spectrophotometry at 650 and 280 nm. The concentration of Alexa647-labeled Rpc34 protein in samples was adjusted to 5 nM by means of 5 mM sodium phosphate pH 8.0 supplemented with 0.05% Tween 20 (MST buffer). The respective ligands—Rpc31(aa 121–251)_Flag, Rpc31(aa 121–251)_Flag_E183K and Rpc31(aa 121–251)_Flag_ΔS151-G164—were also dissolved in MST buffer. The ligand protein samples were subjected to serial dilution to prepare sample concentrations ranging from μM to pM. Each ligand dilution sample was mixed with dye-labeled Rpc34 in a 1:1 volume ratio. After 10-min incubation, the samples were loaded into Monolith™ NT.115 MST Premium Coated capillaries for MST measurements in a Monolith NT.115pico instrument (NanoTemper Technologies) at 25°C. Instrument parameters were adjusted to 40% LED power and medium MST power. Data from three independently pipetted measurements were analyzed using the PALMIST analysis software, and the binding curves were plotted using GUSSI (25,26).

## RESULTS

### Mutational analysis reveals the C-terminal unstructured region of Rpc31 is required for optimal cell growth

We introduced amino acid substitutions and truncations throughout the entire sequence of yeast Rpc31. A list of Rpc31 mutations and associated growth phenotypes are shown in Supplementary Figure S2. Consistent with previous structural and genetic studies, mutations in the N-terminal structured region and the C-terminal Asp-Glu acidic tail resulted in cell growth defects. Surprisingly, we also found that mutations in the unstructured amino acid region from Asp123 to Gly201 impaired cell growth. Multiple sequence alignment revealed that this unstructured region contains a phylogenetically conserved block from Asp123 to Leu136 that is immediately C-terminal to the stalk bridge helix (Figure 1B and Supplementary Figure S3). The functional importance of this conserved block was demonstrated by the cold-sensitive phenotype displayed by yeast strains hosting substitutions of the conserved proline, glutamic acid, phenylalanine, and leucine residues (Figures 1C and Supplementary Figure S3). Although this conserved block has not been resolved in yeast pol III structures, the homologous sequence in human Rpc7 has been resolved as interacting with the WH1 domain of the Rpc3 subunit in the unbound and elongation structures of human pol III (Figures 1B, C, WH1 binding; Supplementary Figure S1) (6,7,18,19).

The sequence from Tyr137 to Gly201 that is C-terminal to the conserved block of Rpc31 is less conserved, yet a series of truncations in this region also yielded yeast strains exhibiting defective cell growth (Figure 1D). We speculate that this region, which is unstructured in the yeast pol III structures, likely serves as a flexible linker to connect the structurally stable N-terminal region and the Asp-Glu acidic tail. Upon inspecting the multiple sequence alignment of this unstructured region, we noticed a mildly conserved sequence block of ∼30 amino acids immediately adjacent to the Asp-Glu acidic tail (Supplementary Figure S3). This sequence block contains multiple conserved leucine residues flanking by charged amino acids, which can be referred to as the pre-acidic region. We denote the remaining amino acid sequence in the C-terminal unstructured region as the linker (Figure 1B and Supplementary Figure S3). Substitutions in the pre-acidic region resulted in the Asp186Ala/Asp187Ala (D186A/D187A) and Glu183Lys (E183K) yeast mutants displaying cold sensitivity (Figure 1D; Supplementary Figure S2), implying that these charged residues exert important functions. Moreover, we detected no apparent cell growth defect upon deleting the first 34 residues of the Rpc31 N-terminus (Supplementary Figure S2). Interestingly, a previous study indicated that methylation of Arg5 and Arg9 of Rpc31 is essential for pol III to maintain optimal transcriptional activity (27). We postulate that downstream RNA processing activities likely compensate for methylation-mediated regulation to sustain homeostasis of pol III-transcribed RNAs and support cell growth.

### The Rpc31 pre-acidic region is important for transcription initiation

**Figure 2.**
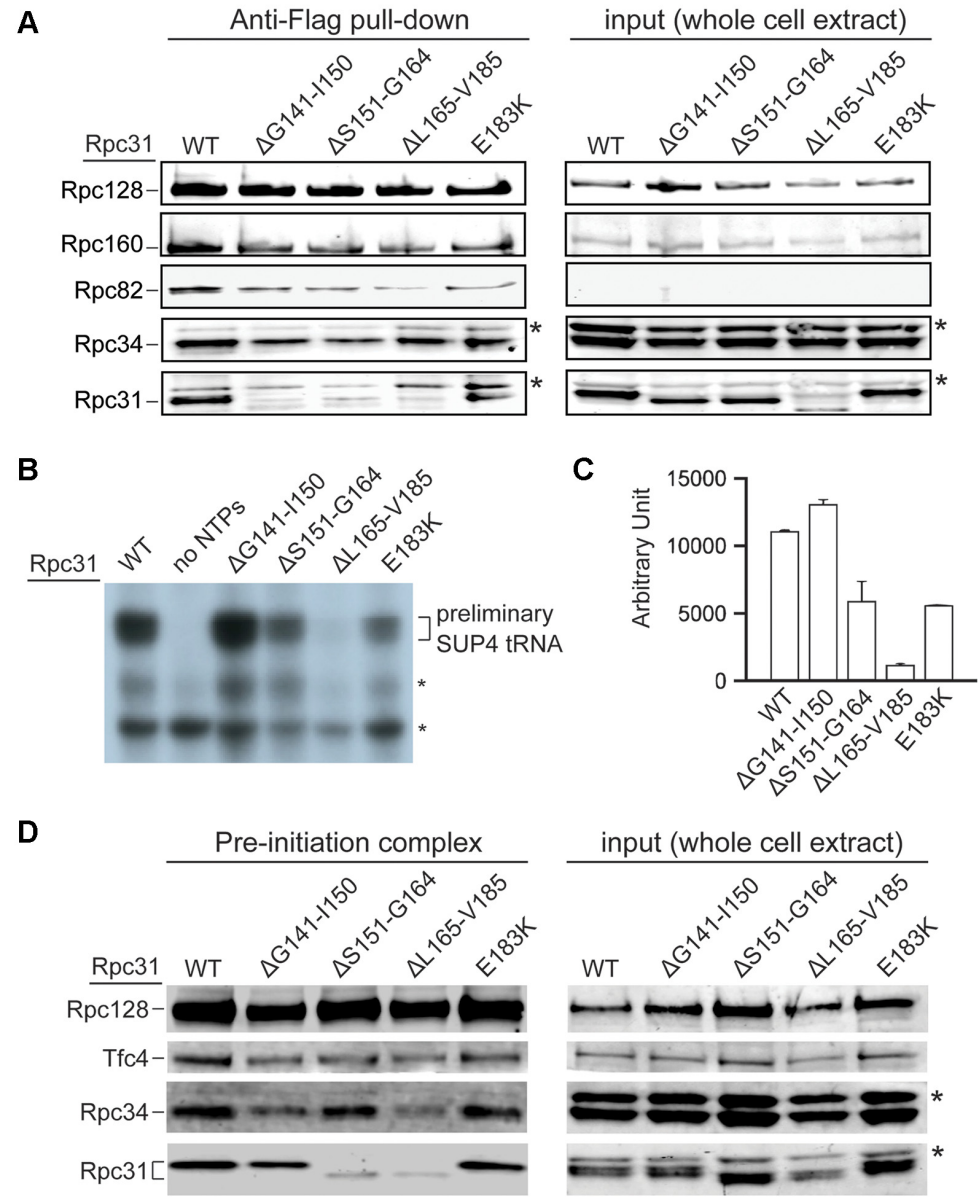
*In vitro* functional analysis of Rpc31 mutants. (**A**) Western blot analysis of pol III co-immunoprecipitation with Rpc31 mutants. The pol III complex was immunoprecipitated through binding of Flag-tagged Rpc128 with anti-Flag agarose beads. Anti-Flag pull-down: Rpc128 and co-immunoprecipitated Rpc160, Rpc82, Rpc34, and Rpc31 were revealed by respective antibodies. Input: proteins in the WCE. * – nonspecific immunostaining gel band. (**B**) *In vitro* transcriptional activity (based on SUP4 tRNA transcripts) of pol III hosting mutant Rpc31. Asterisks indicate non-specific RNAs. (**C**) Quantitation from (B). Error bars indicate SD derived from two independent experiments on separately prepared WCEs. (**D**) Proteins in the pol III PIC from the immobilized template assay. The DNA template contained the SUP4 tRNA gene. Asterisks indicate non-specific immunostaining gel bands.

Our mutational analysis uncovered that the unstructured linker and pre-acidic regions of Rpc31 are required for optimal cell growth. Next, we conducted an immunoprecipitation (IP) analysis on Rpc31 mutants to determine if mutations in these unstructured regions affect protein structural integrity. We utilized anti-Flag antibody beads to immobilize the Flag-epitope-tagged Rpc128 from yeast WCEs in our pulldown experiments. As shown in Figure 2A (Anti-Flag pull-down), Rpc160, Rpc82 and Rpc34 all co-immunoprecipitated, with amounts of precipitated proteins not being affected by Rpc31 mutations. In contrast, truncations of the linker and pre-acidic regions severely affected the levels of Rpc31 that were precipitated. Accordingly, we conclude that stable association of Rpc31 with the pol III complex is compromised by mutations in the C-terminal unstructured linker and pre-acidic regions. Further inspection of relative Rpc31 protein levels in the WCEs indicated that pre-acidic truncation (ΔL165-V185) reduced the respective protein concentration (Figure 2A, input). Thus, pre-acidic truncation also appears to affect Rpc31 protein stability in yeast cells.

To understand if the unstructured region functions in pol III transcription, we conducted *in vitro* transcription assays using WCEs from the mutant strains. As shown in Figures 2B and 2C, except for the Gly141-Ile150 linker truncation, the Ser151-Gly164 linker truncation (ΔS151-G164), the Leu165-Val185 pre-acidic truncation (ΔL165-V185), and the Glu183Lys point mutation (E183K) all severely compromised Sup4 tRNA synthesis. In particular, the Leu165-Val185 pre-acidic truncation elicited the most severe defects in our *in vitro* transcription and cell growth assays (Figures 2B, C and Figure 1D). Next, we utilized the mutant WCEs to analyze PIC formation by means of immobilized template (IMT) assay using DNA containing the SUP4 tRNA gene. As demonstrated in Figure 2D, the ΔS151-G164 and ΔL165-V185 truncation mutants exhibited reduced levels of Rpc31 in the isolated PICs, whereas amounts of the pol III subunits Rpc128 and Rpc34 and the Tfc4 subunit of transcription factor TFIIIC were unaffected. Thus, the linker and pre-acidic peptide regions appear to be critical to supporting stable association of Rpc31 in the PIC and for subsequent SUP4 tRNA transcription. In the case of the ΔL165-V185 pre-acidic truncation variant, the reduced protein level in the PICs could also be attributable to the relatively low protein concentration in the WCE. Interestingly, our IMT analysis also indicates that the Glu183Lys point mutant does not affect stable association of Rpc31 in the isolated PICs, contrasting with the reduced transcriptional activity displayed by this mutant (Figure 2D versus Figures 2B, C) and indicating that residue Glu183 plays a role in a step after PIC formation. Moreover, despite the requirement for stable association of Rpc31 in the pol III complex (Figure 2A), the Gly141-Ile150 linker truncation did not induce any defect in either tRNA synthesis or PIC formation (Figures 2B–D). Thus, we speculate that this linker peptide likely mediates an interaction of Rpc31 in the pol III complex, but this association is dispensable for PIC formation. In summary, our *in vitro* analyses indicate that the Rpc31 linker and pre-acidic regions are important for pol III transcription, and these peptide regions participate both during and after PIC formation.

### Site-directed photo-cross-linking analysis reveals Rpc82 as a major binding target for Rpc31 in the PIC

Previous cryo-EM analyses of initiating and elongating pol III have defined the structural role of the N-terminal region of Rpc31, which acts as a binding module for Rpc82 and the stalk subcomplex. Although structural features of the C-terminal region have yet to be characterized for any functional states, our mutational analysis coupled with *in vitro* assays indicate roles for it in structural integrity and transcription initiation. To investigate if the C-terminal region is involved in protein interactions during transcription initiation, we conducted a site-directed photo-cross-linking analysis to map the protein interactions of Rpc31 in the PIC. First, we incorporated a photo-crosslinking reagent, p-benzyoyl-l-Phenylalanine (BPA), as a non-natural amino acid into yeast Rpc31. BPA was substituted at specific amino acid sites using the suppressor tRNA/tRNA synthetase plasmid system, which targets non-sense TAG codons inserted into the protein coding sequence (22,23). Then, we employed WCEs harboring BPA-containing Rpc31 variants to assemble the PIC using an immobilized DNA template containing the SUP4 tRNA gene. The isolated complexes were UV-irradiated to generate protein crosslinks for subsequent Western blot analysis. This strategy allowed us to identify protein-binding partners for a specific BPA-substituted residue in Rpc31 based on dual antibody-based immunostaining of the cross-linking gel bands.

**Figure 3.**
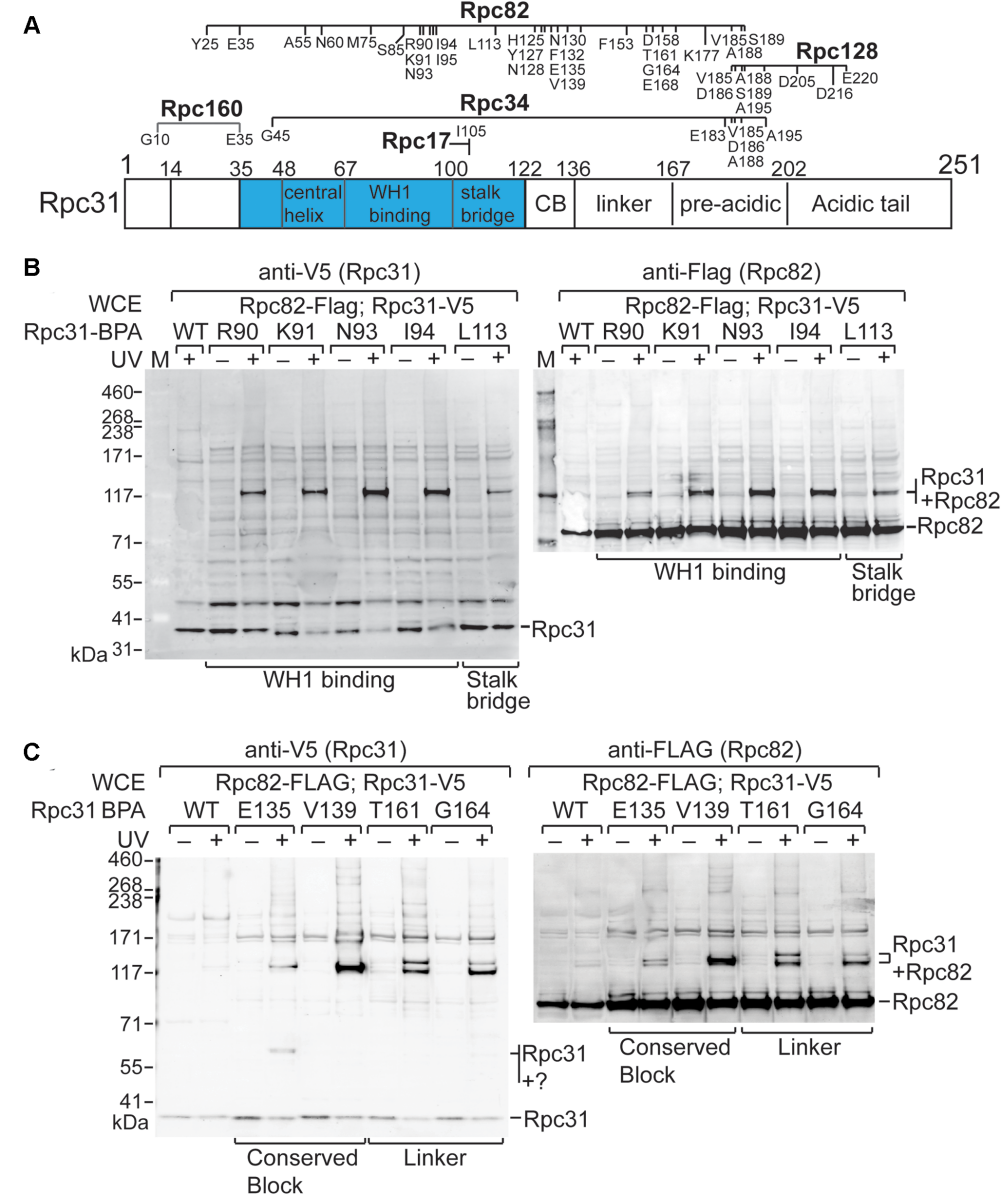
Mapping of protein interactions in isolated PICs. (**A**) Summary of protein interaction mapping by BPA cross-linking. Positions of BPA cross-links are shown above the schematic of Rpc31. (**B**) Rpc31-Rpc82 cross-linking in the WH1-binding and stalk bridge folds of Rpc31. In the Western blot analysis, anti-V5 antibody was utilized to reveal C-terminally V5 epitope-tagged Rpc31, as well as the Rpc31-Rpc82 crosslinks. Rpc31-Rpc82 cross-links were validated by using anti-Flag antibody to probe C-terminally Flag epitope-tagged Rpc82. WCE, whole cell extract. (**C**) Rpc31-Rpc82 cross-linking from BPA positioned in the conserved block and linker regions of Rpc31.

The cross-linking results from our extensive suite of BPA substitutions in Rpc31 are summarized in Figure 3A and Supplementary Table S1. Overall, our data highlight extensive interactions between Rpc31 and Rpc82, consistent with previous structural analyses indicating a strong association between both proteins (6–8). As illustrated in Figure 3B, BPA substitutions illuminated Rpc31-Rpc82 cross-links in the WH1-binding and stalk bridge folds of Rpc31. Furthermore, we inspected the BPA-substituted positions of Rpc31 in the available cryo-EM structure of an initiating pol III (6). Apart from the BPA-substitution at Leu113, all of the Rpc31-Rpc82 cross-links generated from BPA-substituted residues lie within 10 Å (Cα to Cα) of Rpc82 (Supplementary Table S1).

Moreover, the Rpc82 cross-linking sites are in Rpc31 positions that are unstructured in the cryo-EM analyses of pol III (6,7). As shown in Figure 3C and Supplementary Figures S4A, B, positions in both the conserved block and the linker cross-link to Rpc82. This extensive Rpc82 cross-linking with unstructured regions of Rpc31 imply that these regions exert potential structural roles in the PIC. Supporting this hypothesis, the conserved block of the homologous protein in human, Rpc7, has been resolved as binding to the WH1 fold of Rpc3 (a Rpc82 homolog) in the cryo-EM structures of elongating and unbound human pol III complexes (Supplementary Figure S1) (18,19). Given sequence conservation and our cross-linking results, we propose that the conserved block of Rpc31 likely functions as a second binding module to the WH1 domain of Rpc82 in the PIC.

### Cross-linking analysis indicates that the stalk bridge fold of Rpc31 is flexible

**Figure 4.**
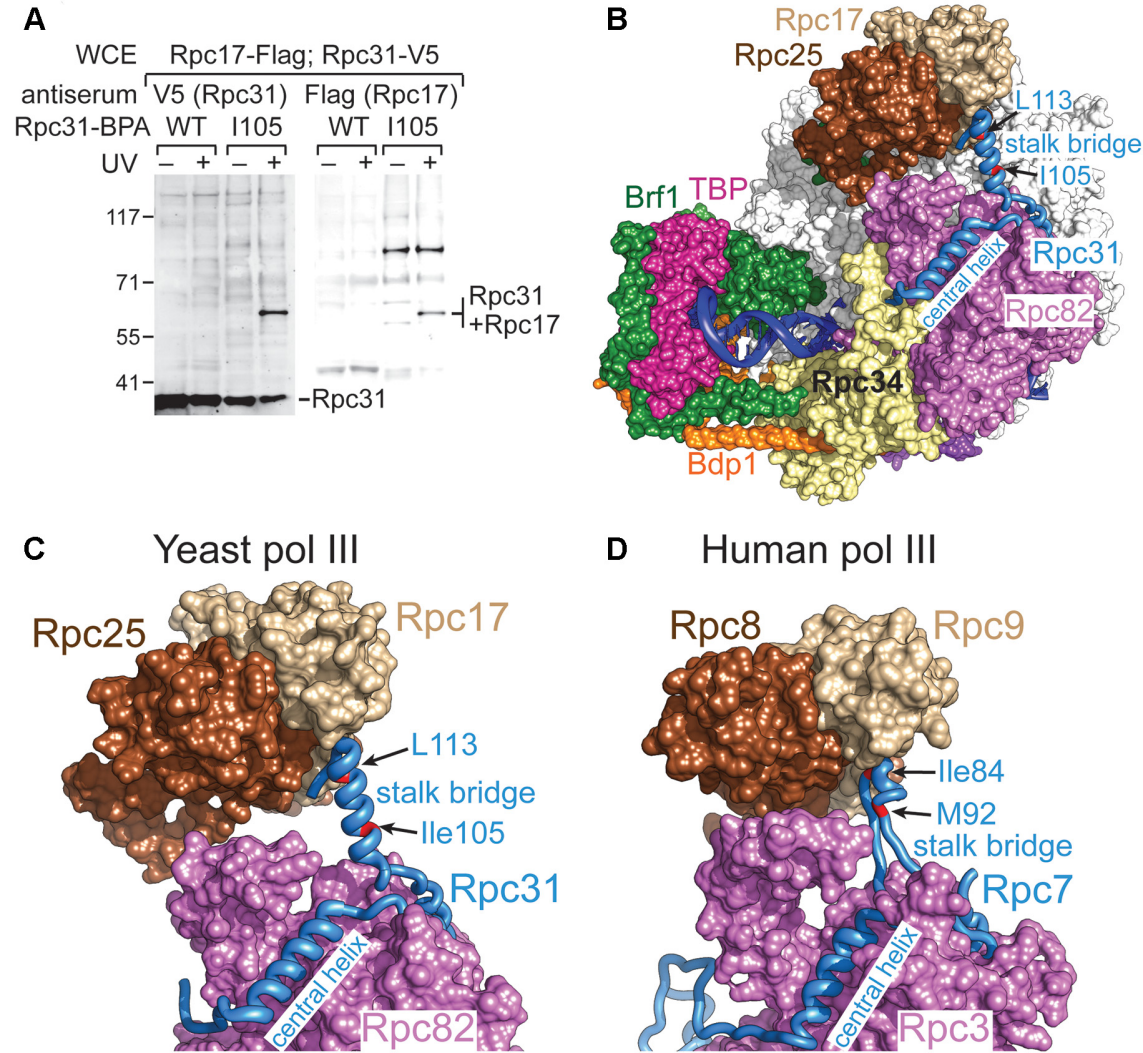
BPA cross-linking analysis of the Rpc31 stalk bridge fold. (**A**) Rpc31-Rpc17 cross-linking. The Western blot analysis indicates that BPA substitution at Ile105 of Rpc31 cross-links to Rpc17. Anti-V5 and anti-Flag antibodies were utilized to reveal the C-terminally V5 epitope-tagged Rpc31 and the C-terminally Flag epitope-tagged Rpc17, respectively. Rpc31-Rpc17 cross-linking was validated through double immunostaining of the fusion protein by anti-V5 and anti-Flag antibodies. (**B**) Positions of Ile105 and Leu113 (in red) in the stalk bridge fold of the Rpc31 backbone trace model (blue). The pol III PIC model is illustrated as in Figure 1A. (**C**) The stalk bridge fold of Rpc31 in the yeast PIC. (**D**) The stalk bridge fold of human Rpc9 (Rpc31 homolog) in the elongating complex of human pol III. The same color scheme for yeast pol III has been applied to the homologous subunits of human pol III. The model is based on the cryo-EM structure of elongating human pol III (PDB code: 7ae1).

In addition to Rpc82, our BPA cross-linking analysis confirms an interaction with the Rpc17 subunit of the stalk subcomplex. As shown in Figure 4A, BPA substitution at Ile105 in the stalk bridge fold of Rpc31 yielded a cross-link with Rpc17. Inspecting the cryo-EM structure of an initiating pol III complex generated by Abascal-Palacios *et al.* (Figures 4B, C) (6), we observed that Ile105 is located at least 20 Å (Cα to Cα) from any nearby amino acids of Rpc17. In addition, as mentioned above, we detected Rpc31-Rpc82 cross-linking at another stalk bridge residue, Leu113, which is positioned ∼20 Å from Rpc82 (Figures 4B, C). Given that the benzophenone functional group of BPA attracts a hydrogen atom from a C-H bond upon UV irradiation to create a covalent C-C bond, it can only link amino acids within distances of approximately 10 Å (Cα to Cα) (28). Therefore, our observations of Ile105 cross-linking with Rpc17 and Leu113 cross-linking with Rpc82 do not conform to the cryo-EM structure of an initiating pol III complex. We surmise that the stalk bridge fold of Rpc31 is structurally flexible, thereby permitting long-range cross-linking. In support of this hypothesis, the stalk bridge fold in the structure of the initiating pol III complex was built with only a backbone model, lacking sidechain details (6). Interestingly, in the structures of elongating and unbound human pol III (18), the stalk bridge fold of Rpc7 (a Rpc31 homolog) was assigned a different conformation to allow residue Ile84 of Rpc7 (homologous to Ile105) to be in direct contact with Rpc9 (the Rpc17 homolog) (compare Figure 4C, D; Supplementary Figure S3B). Accordingly, our BPA cross-linking data imply structural flexibility and potentially alternative structural arrangements for the stalk bridge of Rpc31.

### Cross-linking analysis reveals the Rpc31 N-terminus binds with the polymerase core within the PIC

**Figure 5.**
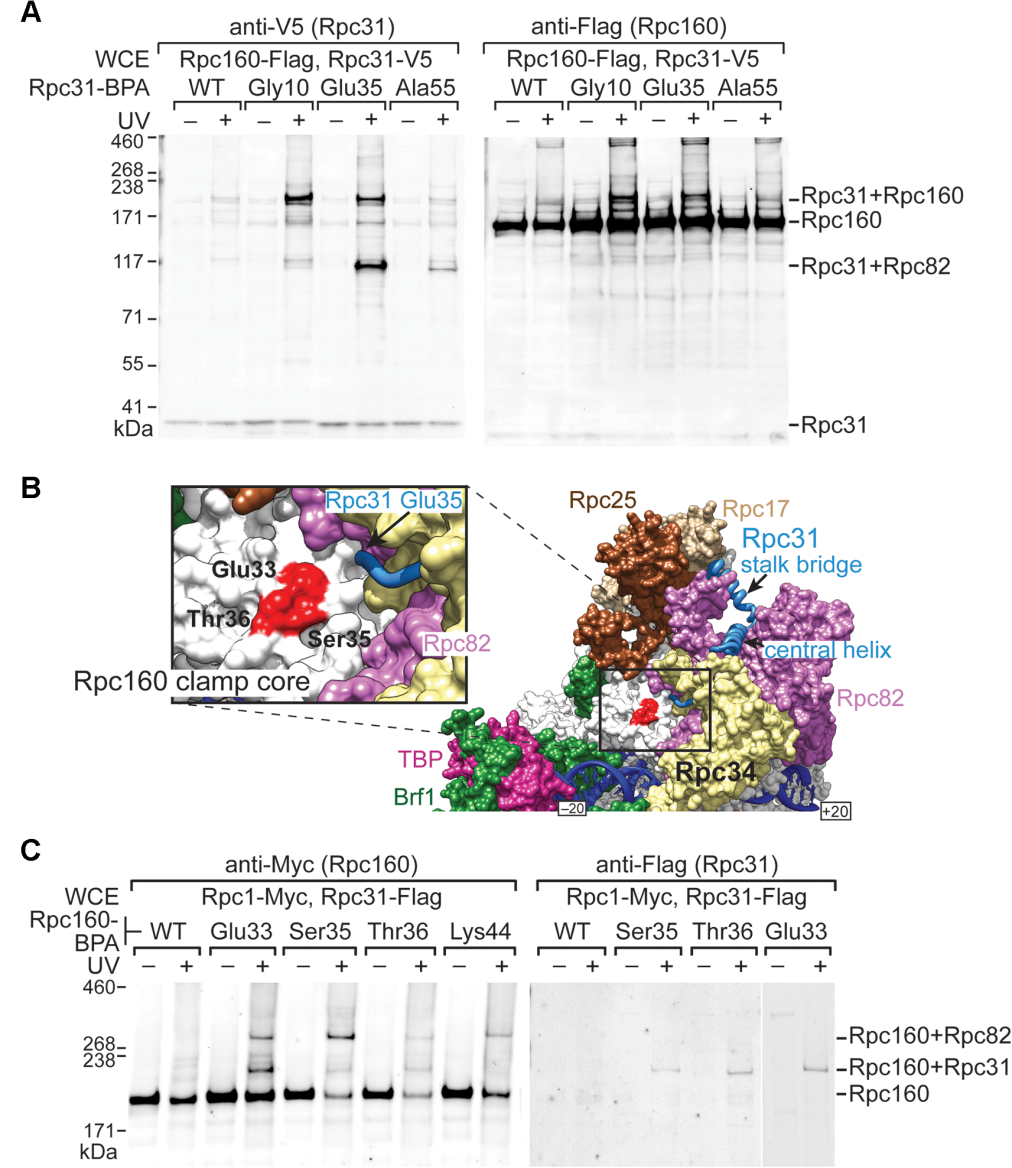
Rpc31 N-terminus interacts with the clamp core domain of pol III. (**A**) Rpc31-Rpc160 cross-linking. The Western blot analysis indicates BPA substitutions at Gly10 and Glu35 of Rpc31 cross-link to Rpc160. Anti-V5 and anti-Flag antibodies were utilized to reveal C-terminally V5 epitope-tagged Rpc31 and C-terminally Flag epitope-tagged Rpc160, respectively. The Rpc31-Rpc160 cross-links were validated through double immunostaining of the fusion protein gel bands using anti-V5 and anti-Flag antibodies. These two positions also cross-link to Rpc82. (**B**) Positions of BPA-substituted residues involved in Rpc31-Rpc160 cross-linking. The pol III PIC model is shown (PDB code: 6eu0). Residues highlighted in red are in the clamp core of Rpc160 that cross-links with Rpc31. Glu35 of Rpc31 is indicated. (**C**) Rpc160-Rpc31 cross-linking. The Western blot analysis indicates that BPA substitutions at Glu33, Ser35, and Thr36 of Rpc160 cross-link to Rpc31. The positions of these three residues are highlighted in (B). Anti-Myc and anti-Flag antibodies were utilized to reveal C-terminally Myc epitope-tagged Rpc160 and C-terminally Flag epitope-tagged Rpc31, respectively. These positions also cross-link to Rpc82.

In cryo-EM structures of an initiating pol III complex, the first 34 amino acids of Rpc31 are unstructured (6,7). In contrast, in the respective structures of unbound and elongating human pol III, the N-terminal region of homologous Rpc7α is bound to the clamp core of the catalytic subunit Rpc1 (Supplementary Figures S1&3B) (18,19). Given their sequence conservation (Supplementary Figure S3B), we wondered if, like human Rpc7α, the yeast Rpc31 N-terminus also interacts with the clamp core. Therefore, we incorporated BPA into the Rpc31 N-terminus to explore its protein-binding targets within the PIC. As demonstrated in Figure 5A, BPA replacements of Gly10 and Glu35 generated cross-links with catalytic subunit Rpc160 of yeast pol III. We also detected a second cross-link between Glu35 of Rpc31 and Rpc82, consistent with Glu35 being positioned close to Rpc82 in the initiating pol III complex (Figure 5B). To further explore if Rpc31 interacts with the clamp core region of Rpc160, we conducted a reciprocal experiment by incorporating BPA into Rpc160. As displayed in the structure of initiating pol III (Figure 5B) and our cross-linking analysis (Figure 5C), BPA replacements of surface residues Glu33, Ser35 and Thr36 of the Rpc160 clamp core generated cross-links with Rpc31. Together, our cross-linking data for Rpc160 and Rpc31 reveal that the Rpc31 N-terminus is positioned on the clamp core region of Rpc160, similar to the binding mode of homologous Rpc7α and Rpc1 in the human pol III complex.

### The pre-acidic region of Rpc31 interacts with Rpc34, Rpc82 and Rpc128 in the PIC

**Figure 6.**
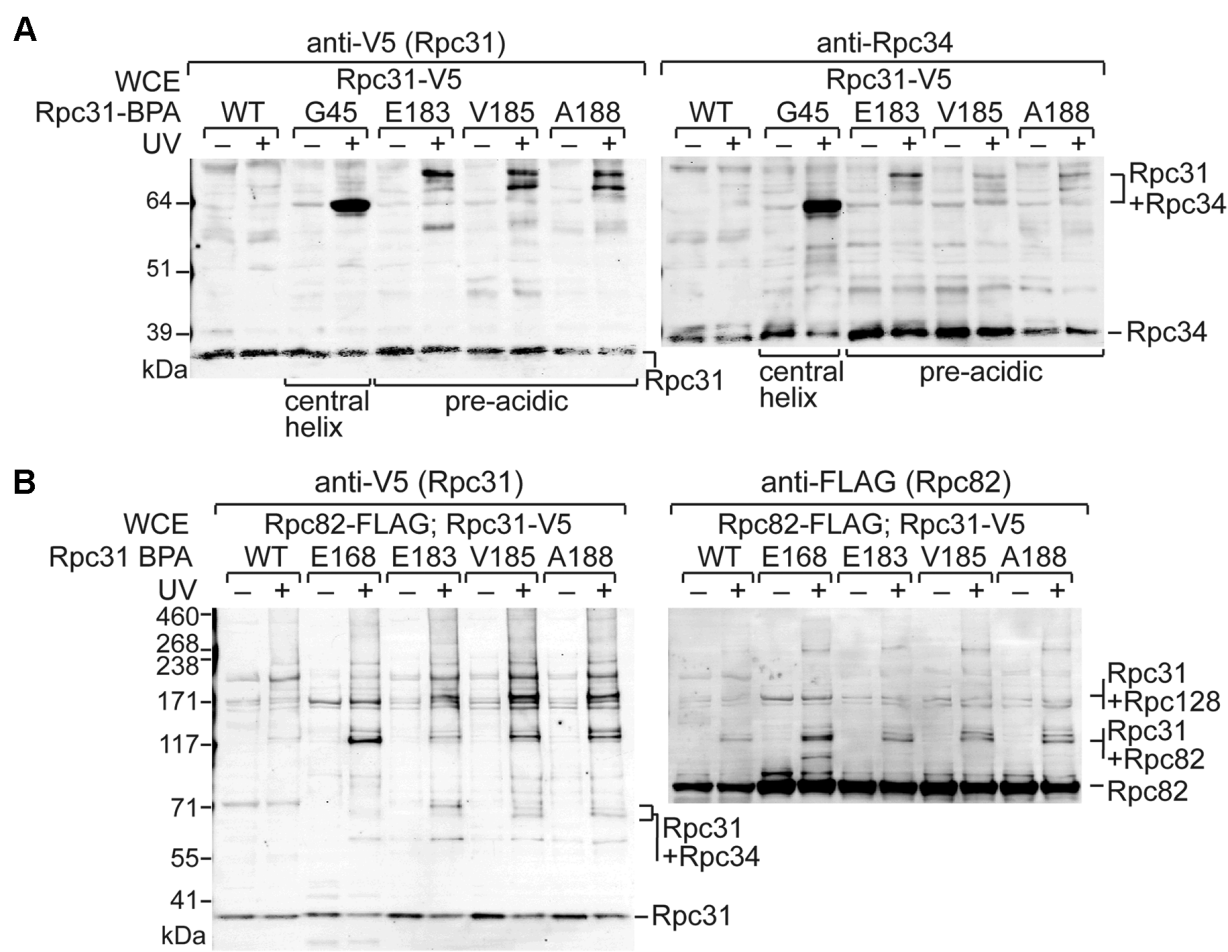
The pre-acidic region of Rpc31 interacts with Rpc34 and Rpc82 in the PIC. (**A**) Rpc31–Rpc34 cross-linking. The Western blot analysis indicates that BPA substitutions in the central helix and the pre-acidic region of Rpc31 cross-link to Rpc34 in the isolated PICs. Anti-V5 and anti-Rpc34 antibodies were utilized to reveal the Rpc31–Rpc34 cross-links. (**B**) Rpc31-Rpc82 cross-linking. BPA substitutions in the pre-acidic region yield Rpc31-Rpc82 cross-links, as confirmed by immunostaining with the indicated antibodies.

Although cryo-EM of the initiating pol III complex has uncovered some unstructured regions, here we have provided functional analyses indicating that the pre-acidic region of Rpc31 is an important peptide sequence for pol III transcription. By incorporating BPA into that sequence to illuminate cross-linking in the PIC, we have uncovered that it interacts with Rpc34, Rpc82 and Rpc128 (as summarized in Figure 3A; detailed in Figure 6A, B, and Supplementary Figure S5A). Moreover, we found that Gly45 of Rpc31 crosslinks with Rpc34 near its N-terminal central helix (Figure 6A), which is consistent with positioning of Gly45 in the vicinity of Rpc34 in the initiating pol III complex, as illustrated in Supplementary Figure S5B.

In addition to our BPA-cross-linking analysis, a previous lysine/lysine cross-linking/mass spectrometric analysis comprehensively mapped pol III protein interactions (12). The DSS cross-linker used in lysine-specific cross-linking analyses is more flexible than BPA, enabling a greater reaction distance of up to 20 Å (Cα to Cα). Furthermore, unlike for our BPA cross-linking analysis on the pol III PIC, the previous lysine-specific cross-linking analysis was performed using purified unbound pol III. By comparing these two cross-linking datasets, we found different patterns of protein interactions within the pre-acidic region of Rpc31 (Supplementary Figure S6A). Whereas Lys179 and Lys196 in the pre-acidic region respectively cross-linked with Rpc82/ABC27 and Rpc160, BPA substitutions in the same region yielded cross-links with Rpc34, Rpc82 and Rpc128. As illustrated in Supplementary Figure S6B, the cross-linked lysine residues—including Lys143 of Rpc160, Lys242 of Rpc82, and Lys94 of ABC27 (Rpb5)—are clustered near the downstream duplex DNA, implying that the pre-acidic region likely adopts a position at the DNA entry surface of the pol III active site tunnel. This lysine-derived localization also enables loading of the Asp-Glu acidic tail into the active site tunnel, i.e. similar to the structural position of the homologous region in the unbound human pol III (Supplementary Figures S1A, C). In contrast, our BPA-based cross-linking revealed a potential alternative localization for the pre-acidic region, particularly considering our finding of an interaction of the pre-acidic region with Rpc34. Given that Rpc34 is localized on the DNA exit surface of the active site tunnel (Figure 1A and Supplementary Figure S6B), we postulate that the pre-acidic region shifts away from the DNA entry surface to contact Rpc34. Thus, our cross-linking data reveals a possible positional switch for the pre-acidic region upon PIC formation from the unbound pol III.

### The pre-acidic region of Rpc31 interacts with Rpc34 in a sequence-dependent manner

**Figure 7.**
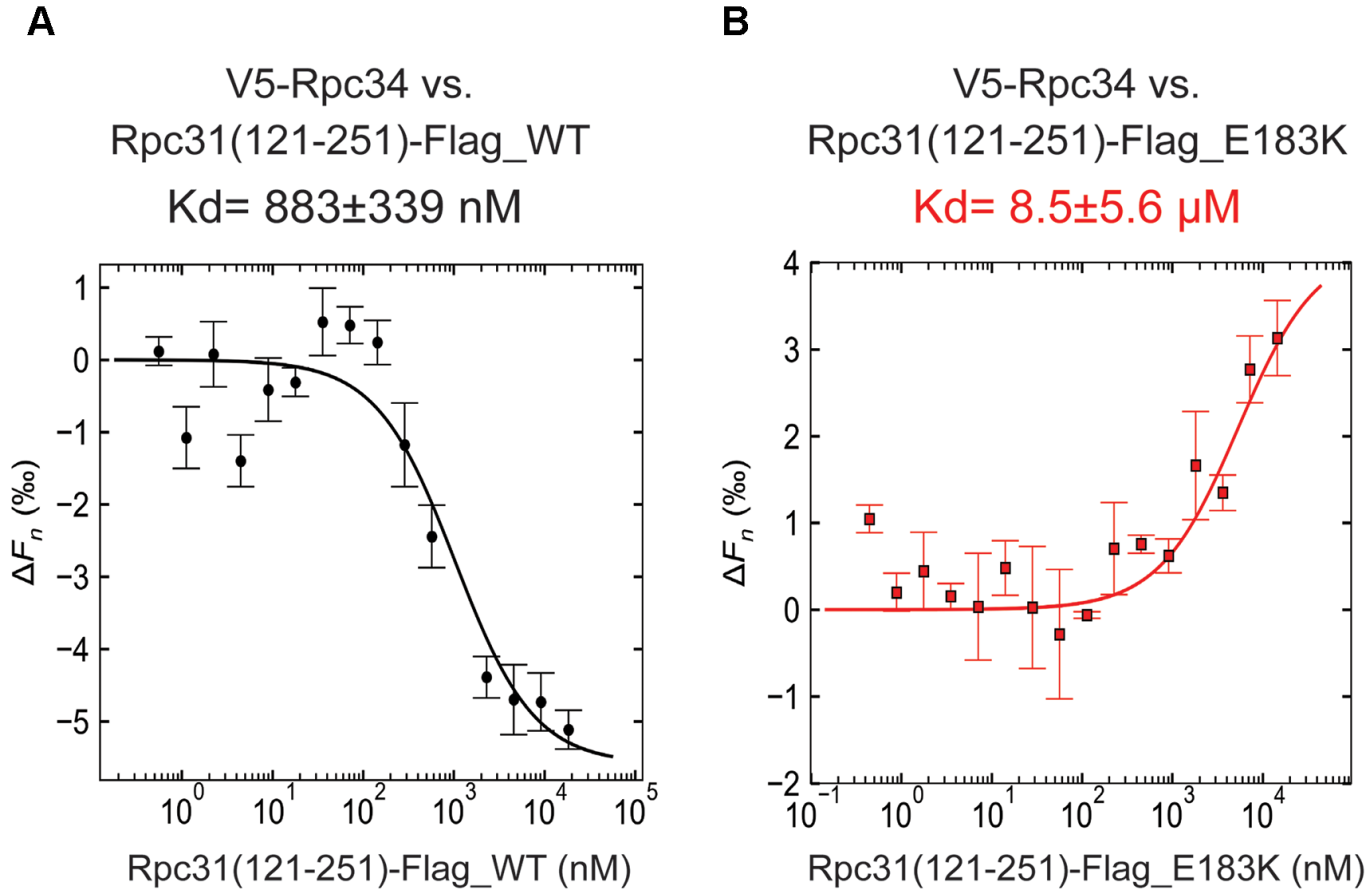
Determination of the dissociation constant between Rpc31 (aa 121–251) peptide and Rpc34. (**A**) MST binding curve for the interaction between wild-type (WT) Rpc31 (aa 121–251) and Rpc34. For the MST experiment, the concentration of Alexa 647-labeled Rpc34 was kept constant (5 nM), whereas the concentration of the non-labeled binding partner, i.e. Rpc31 (aa 121–251), varied from 1.16 μM to 0.000035 μM. A MST-on time of 5 s was used for this analysis, giving a *K*_d_ of 883 ± 339 nM (*n* = 3 independent measurements, error bars represent the standard deviation). (**B**) MST binding curves for Rpc31 (aa 121–251) peptide interactions with wild-type (WT) and Glu183Lys point-mutated Rpc34. MST assay conditions were as in (A), and the *K*_d_ value is 8.5 ± 5.6 μM.

Our BPA cross-linking analysis has shown that the C-terminal unstructured region of Rpc31 is involved in specific protein interactions within the PIC. Notably, we have defined an interaction between the pre-acidic region of Rpc31 and Rpc34 that likely reflects a positional switch for the former's C-terminal region away from the downstream DNA entry surface towards the upstream DNA exit surface of the pol III active site tunnel. To further characterize this interaction, we carried out a pairwise interaction analysis by microscale thermophoresis (MST) to quantitate the binding affinities of a series of recombinantly expressed and purified Rpc31 C-terminal wild-type and mutant peptides and fluorescently-labeled Rpc34 protein (Supplementary Figure S7A). As demonstrated in Figure 7A, our MST data indicates that the Rpc31 C-terminal Ser121-Phe251 peptide and Rpc34 interact with a dissociation constant (*K*_d_) of 883 nM. An Rpc31 peptide with a Glu183Lys (E183K) point mutation in the pre-acidic region exhibited weaker binding with Rpc34 (*K*_d_ = 8.5 μM; Figure 7B). Similar analyses on mutant Rpc31 peptides with truncations in the C-terminal region exhibited similarly weak binding affinities with Rpc34, generating Kd values ranging from 1.2 to 6.1 μM (Supplementary Figures S7B–D). In summary, our MST analysis supports a specific Rpc34 interaction for the C-terminal unstructured region of Rpc31 and, importantly, the Rpc31–Rpc34 interaction depends on the amino acid sequence of the pre-acidic region.

## DISCUSSION

In this study, we applied mutational, biochemical, and biophysical analyses to provide new insights into the roles of Rpc31 peptide regions in cell viability and pol III transcription activity. Notably, our site-directed photo-cross-linking analyses support a stable structural association between the Rpc31 N-terminal region and Rpc82. Our data also indicate structural flexibility of the Rpc31 stalk bridge and N-terminal binding to the clamp core of Rpc160. Our cross-linking analyses further reveal the interactome of the C-terminal region that was unresolved by previous structural studies. Apart from the tight association with Rpc82 protein, we found that this C-terminal region interacts with Rpc34 and Rpc128. We mapped this Rpc31–Rpc34 interaction to the pre-acidic region of Rpc31, and an MST assay revealed it to be sequence-dependent. Thus, we have added to the suite of known Rpc31 protein interactions, which comprise both stable and flexible interactions within the unbound pol III complex as well as in the PIC.

Our cross-linking analysis supports that the Rpc31 N-terminus interacts with the clamp core of the Rpc160 catalytic subunit within the PIC. However, the interaction appears to be rather unstable, as the Rpc31 N-terminus was not resolved as contacting the clamp core in all available unbound, elongating, and initiating yeast pol III structures (5–8,29,30). In recent structural analyses of human pol III by the Müller group, the Rpc31 homologous protein Rpc7α was reported to utilize residue Tyr12 to establish critical aromatic stacking and hydrogen bonding with amino acids in the coiled-coil fold of the clamp core (18). As the pol III repressor Maf1 also interacts with the clamp coiled-coil (30), the Rpc7α N-terminus was suggested to abrogate the repressive action by Maf1. In contrast, the other Rpc31 homolog (Rpc7β) lacks the Tyr12 residue, and the Rpc7β N-terminus was hypothesized to bind less tightly to the clamp core. Furthermore, the Müller group also found that the amino acid sequence of the Rpc31 N-terminus is more similar to that of Rpc7β. Consequently, the Rpc31 N-terminus is not likely to establish strong binding with the clamp core, as evidenced by the lack of a resolved structure in pol III complexes. Despite remaining unresolved in various structural analyses, our study provides strong evidence for interaction between the Rpc31 N-terminus and the clamp core in the PIC. We postulate that the Rpc31 N-terminus not only competes with Maf1 to modulate de-repression of pol III transcription, but also supports stable PIC formation through its interaction with the clamp core.

Despite a previous yeast two-hybrid analysis also uncovering the Rpc31–Rpc34 interaction, it has not been observed in mass spectrometric analyses of the purified pol III complex (31–33). In those mass spectrometric studies, no Rpc31–Rpc34 dimer was observed and instead Rpc82 was found to serve as a bridging subunit for Rpc31 and Rpc34 in the Rpc82/34/31 trimeric subcomplex. Rpc34 was also found to dissociate more readily from the trimer and from the pol III complex. Furthermore, recent lysine-lysine cross-linking/mass spectrometric analysis on purified pol III found no evidence of an interaction between Rpc31 and Rpc34. Thus, previous studies have not characterized a stable Rpc31–Rpc34 interaction.

**Figure 8.**
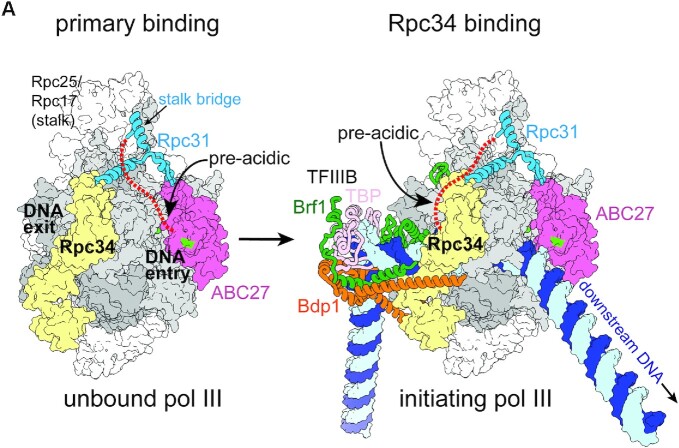
The unbound and initiating pol III models are based on the cryo-EM structure of an initially transcribing complex (PDB code: 6eu0). Rpc34 and ABC27 are colored yellow and pale magenta, respectively. Rpc160 and Rpc128 are colored dark and light gray, respectively. The remaining subunits of pol III are colored white. Rpc31 is represented by the blue backbone trace model. The red dashed line extending from the stalk bridge fold of Rpc31 indicates the location of the pre-acidic region. The DNA entry and exit surfaces are indicated in the unbound pol III. The pre-acidic region of Rpc31 resides on the DNA entry surface of the unbound pol III that contacts ABC27 and Rpc160. Green patches on the DNA entry surface represent lysine residues involved in cross-linking with the pre-acidic region of Rpc31 in the purified (unbound) pol III. In the initiating pol III, Brf1, Bdp1 and TBP are shown as the backbone trace representation and are colored separately, as indicated. The template and non-template DNA strands are colored blue and cyan, respectively. Relative to the unbound pol III, the pre-acidic region switches its interaction to Rpc34 upon formation of the initiating complex.

Our study shows that the pre-acidic region functions in pol III transcription. The pre-acidic region interacts with Rpc34 at a position near the DNA exit surface of pol III. This PIC-specific interaction contrasts with a localization on the DNA entry surface of pol III derived from a lysine-lysine cross-linking analysis of unbound pol III (12). Given that our MST analysis demonstrated that the pre-acidic region possesses a mild affinity for Rpc34 (*K*_d_ of 883 nM), this region likely interacts suboptimally with Rpc34 that has relocated from its primary binding site on the DNA entry surface. We propose that this positional switch is an important mechanism by which the Rpc31 C-terminus regulates pol III functional states. As illustrated in Figure 8, in its primary binding mode, the pre-acidic region is located at the DNA entry surface of the active site tunnel of pol III. This positioning also supports loading of the Rpc31 Asp-Glu acidic tail into the pol III active site tunnel, as partially revealed in the structure of unbound human pol III (Supplementary Figure S1). As proposed previously, binding of this acidic tail at the active site prevents pol III from interacting non-specifically with genomic DNA (14,19). Upon pol III recruitment by the transcription factor TFIIIB (the Brf1/Bdp1/TBP complex; Figure 8), the DNA is loaded into the active site tunnel, displacing the Asp-Glu acidic tail and thereby enabling binding of the pre-acidic region to Rpc34 for stable association of Rpc31 within the PIC. In summary, our study reveals that the pre-acidic region of Rpc31 acts as a flexible structural motif to regulate both the unbound state and transcription initiation of pol III.

## DATA AVAILABILITY

The data underlying this article are available in the article and in its online supplementary material.

## Supplementary Material

gkac1163_Supplemental_File
