# Supplementary material for: Site-directed biochemical analyses reveal that the switchable C-terminus of Rpc31 contributes to RNA polymerase III transcription initiation
Source: Nucleic Acids Res. 2022 Dec 9;51(9):4223–36. doi: 10.1093/nar/gkac1163 (PMC10201443; doi:10.1093/nar/gkac1163)
Supplement: gkac1163_Supplemental_File [file gkac1163_supplemental_file.pdf]

Supplementary Materials

Site-directed Biochemical Analyses Reveal that the Switchable C-terminus of Rpc31 Contributes to RNA Polymerase III Transcription Initiation

Arvind Chandra Shekhar, Yuan-En Sun, Seok-Kooi Khoo, Yu-Chun Lin, Ester Betaria Malau, Wei-Hau Chang, and Hung-Ta Chen

Supplementary Figures S1-S7

Supplementary Table S1

Supplementary Figure S1

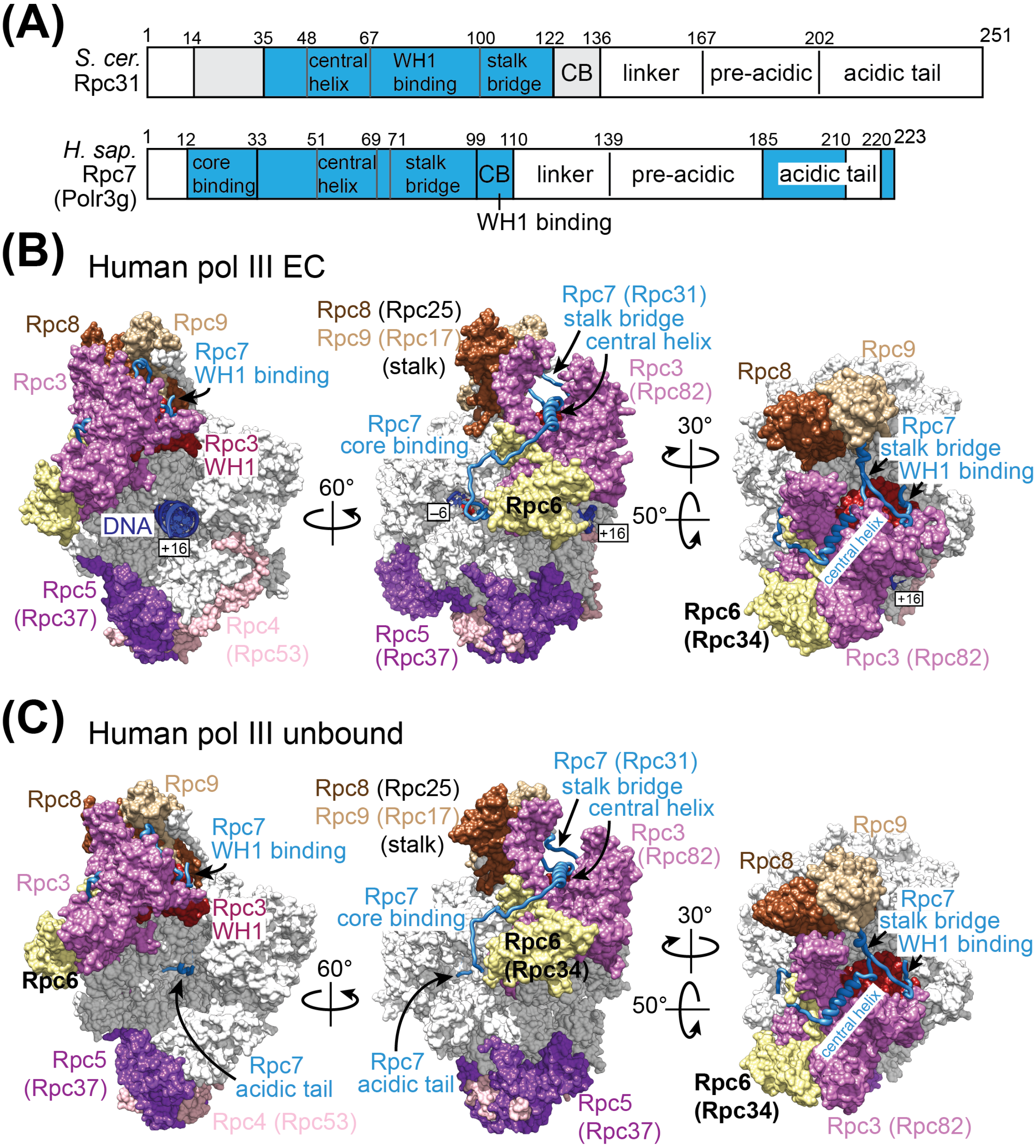

Supplementary Figure S1. (A) Structural regions in S. cerevisiae Rpc31 and human Rpc7. **(B)** Cryo-EM structural model of human pol III in the elongating state. The elongating complex of human pol III is shown as the molecular surface model. The 12-subunit core is colored white, and pol III-specific subunits are separately colored with the same color scheme for their homologous subunits in the yeast pol III in Fig. 1A. Rpc7 (Rpc31 homolog) is shown with the blue backbone trace model. Three orientations are provided. DNA is in dark blue. RNA (red) is located inside the active site. The PDB code is 7ae1 (1). **(C)** Cryo-EM structural model of the human pol III unbound complex (PDB code: 7d59) (2). The position of the Rpc7 acidic tail is indicated.

Supplementary Figure S2

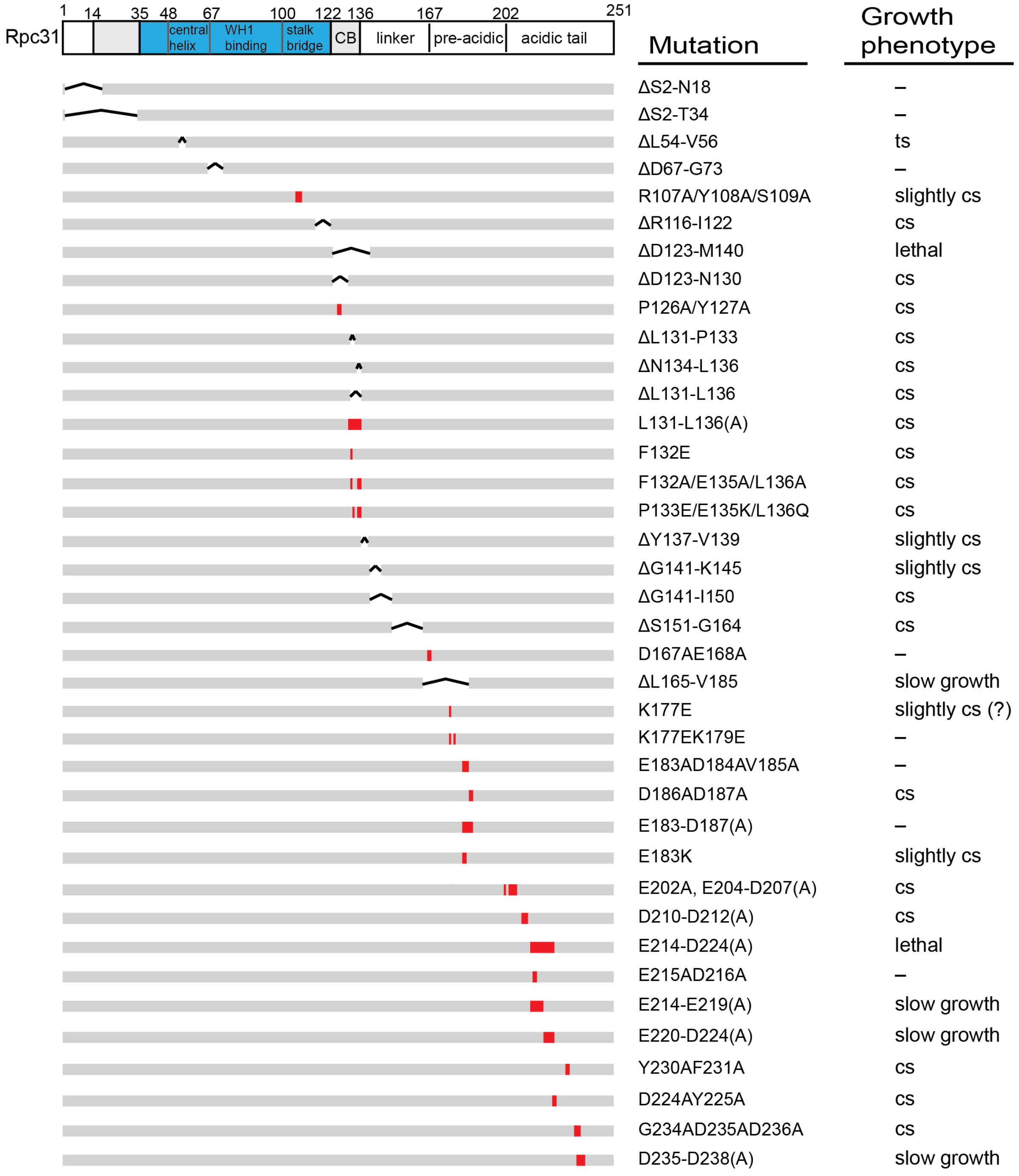

Supplementary Figure S2. Rpc31 mutational analysis. Deletions and amino acid substitutions of *S. cerevisiae* Rpc31 are as indicated. ts: temperature sensitive cell growth at 35 °C. cs: cold sensitive cell growth at 16 °C. slow growth: slow cell growth at all temperatures (16, 25, 30, and 35 °C) tested. Gray colored boxes are unaltered sequences. Deleted amino acid segments are indicated by the connecting lines. Amino acid substitutions are colored in red. (A), multiple alanine replacement in the amino acid stretch. Δ, deletion of the amino acid stretch.

Supplementary Figure S3

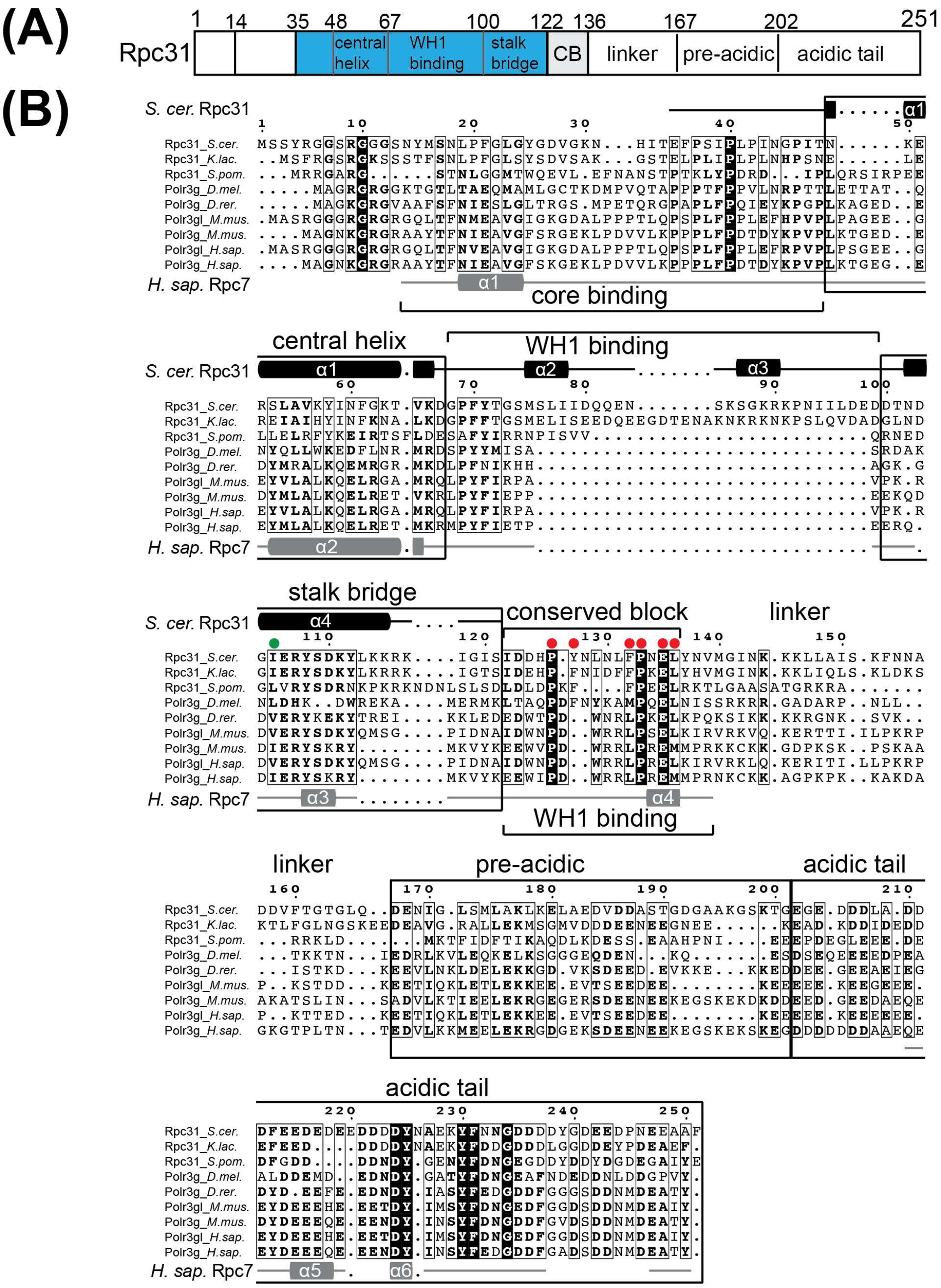

Supplementary Figure S3. Rpc31 multiple sequence alignment. **(A)** Structural regions of Rpc31. **(B)** Multiple sequence alignment. Structurally resolved α-helices of S. cerevisiae Rpc31 and human Rpc7 (H. sapiens Polr3g) are indicated by black and gray boxes listed above and below the alignment, respectively. The sequence alignment is generated using the ClustalX and ESPript programs. Accession number: *S. cerevisiae* (baker's yeast) Rpc31, Uniprot P17890; *K. lactis* (*Kluyveromyces* yeast) Rpc31, GenBank QEU58720; *S. pombe* (fission yeast) Rpc31, Uniprot Q8WZJ8; *D. melanogaster* (Fruit fly) Polr3g, Uniprot Q86BI8; *D. rerio* (Zebrafish) Polr3g, GenPept XP_021331627; *M. musculus* (mouse) Polr3gl, Uniprot Q8R0C0; *M. musculus* Polr3g, Uniprot Q6NXY9; *H. sapiens* Polr3gl (human Rpc32β), Uniprot Q9BT43; *H. sapiens* Polr3g (human Rpc32α), Uniprot O15318. Green dot indicates Ile105 of Rpc31. Red dots indicate the mutated residues in the conserved block of Rpc31. The alignment was generated by the Clustal W and ESPript programs (3,4).

Supplementary Figure S4

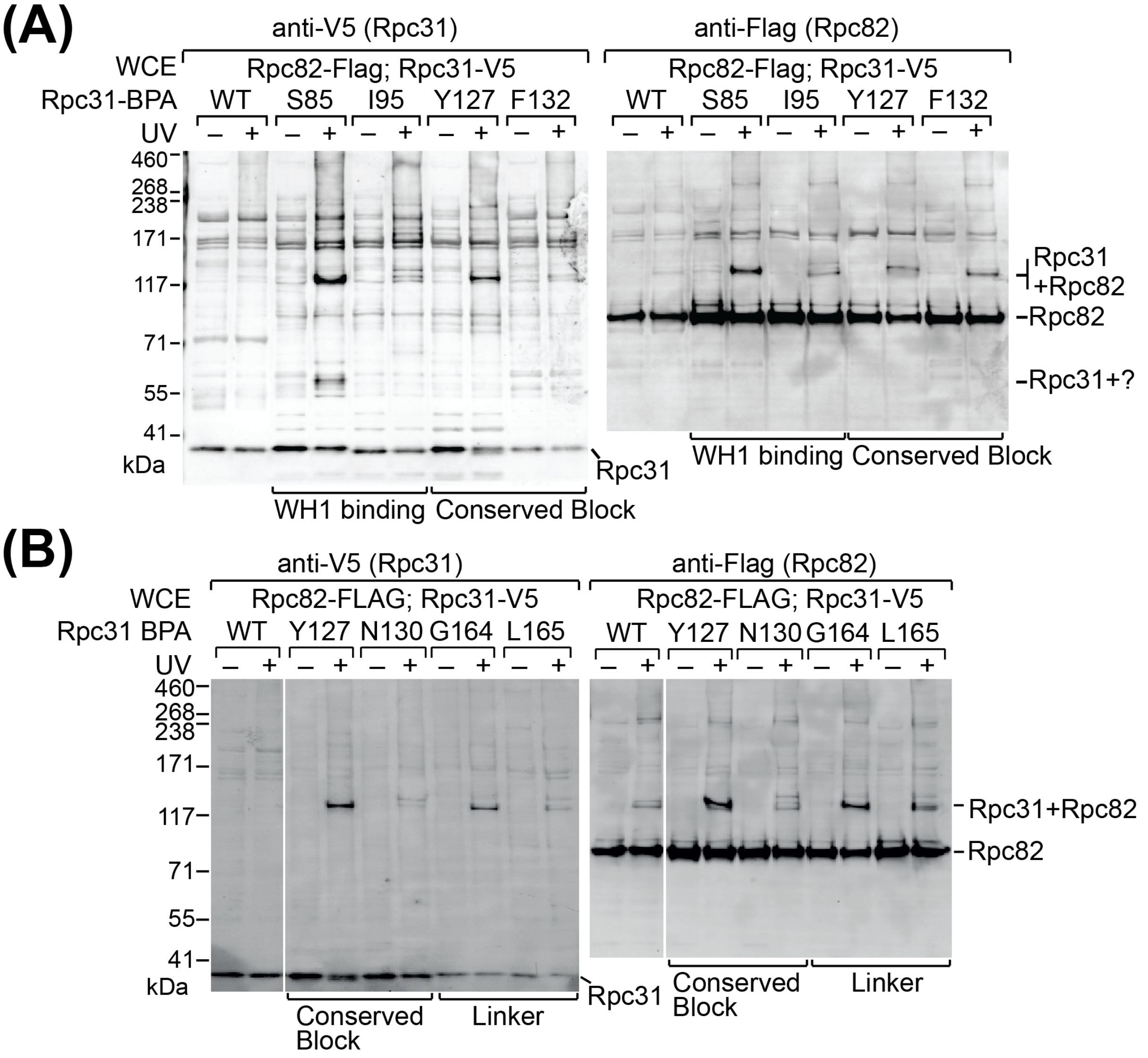

Supplementary Figure S4. (A) Rpc31-Rpc82 cross-linking from BPA positioned in the WH1-binding and conserved block of Rpc31. In the Western analysis of BPA cross-linking, the anti-V5 antibody was utilized to reveal the C-terminally V5 epitope-tagged Rpc31, as well as the Rpc31-Rpc82 crosslinks. The Rpc31-Rpc82 cross-links were validated by using anti-Flag antibody to probe the C-terminally Flag epitope-tagged Rpc82. WCE, whole cell extract. **(B)** Rpc31-Rpc82 cross-linking from BPA positioned in the conserved block and linker regions of Rpc31.

Supplementary Figure S5

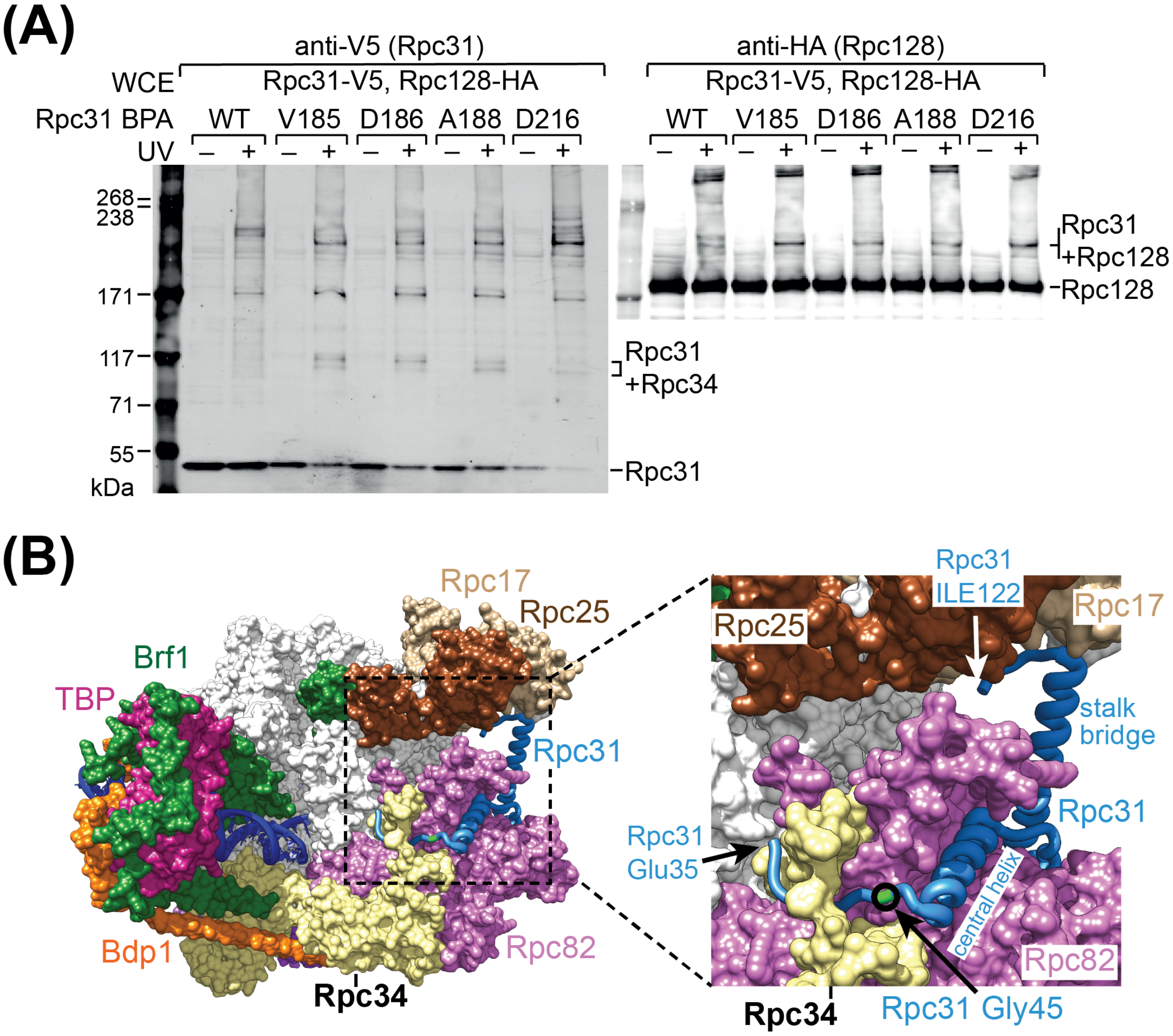

Supplementary Figure S5. (A) Rpc31-Rpc128 cross-linking from BPA positioned in the pre-acidic region of Rpc31. In the Western analysis of BPA cross-linking, the anti-V5 antibody was utilized to reveal the C-terminally V5 epitope-tagged Rpc31, as well as the Rpc31-Rpc82 crosslinks. The Rpc31-Rpc128 cross-links were validated by using anti-HA antibody to probe the C-terminally HA epitope-tagged Rpc128. WCE, whole cell extract. **(B)** Position of Gly45 of Rpc31 in the pol III PIC. The structural model of pol III PIC is illustrated as in Fig. 1A. The position of Gly45 (circled site on the backbone trace model) can be better visualized in the enlarged view on the right.

Supplementary Figure S6

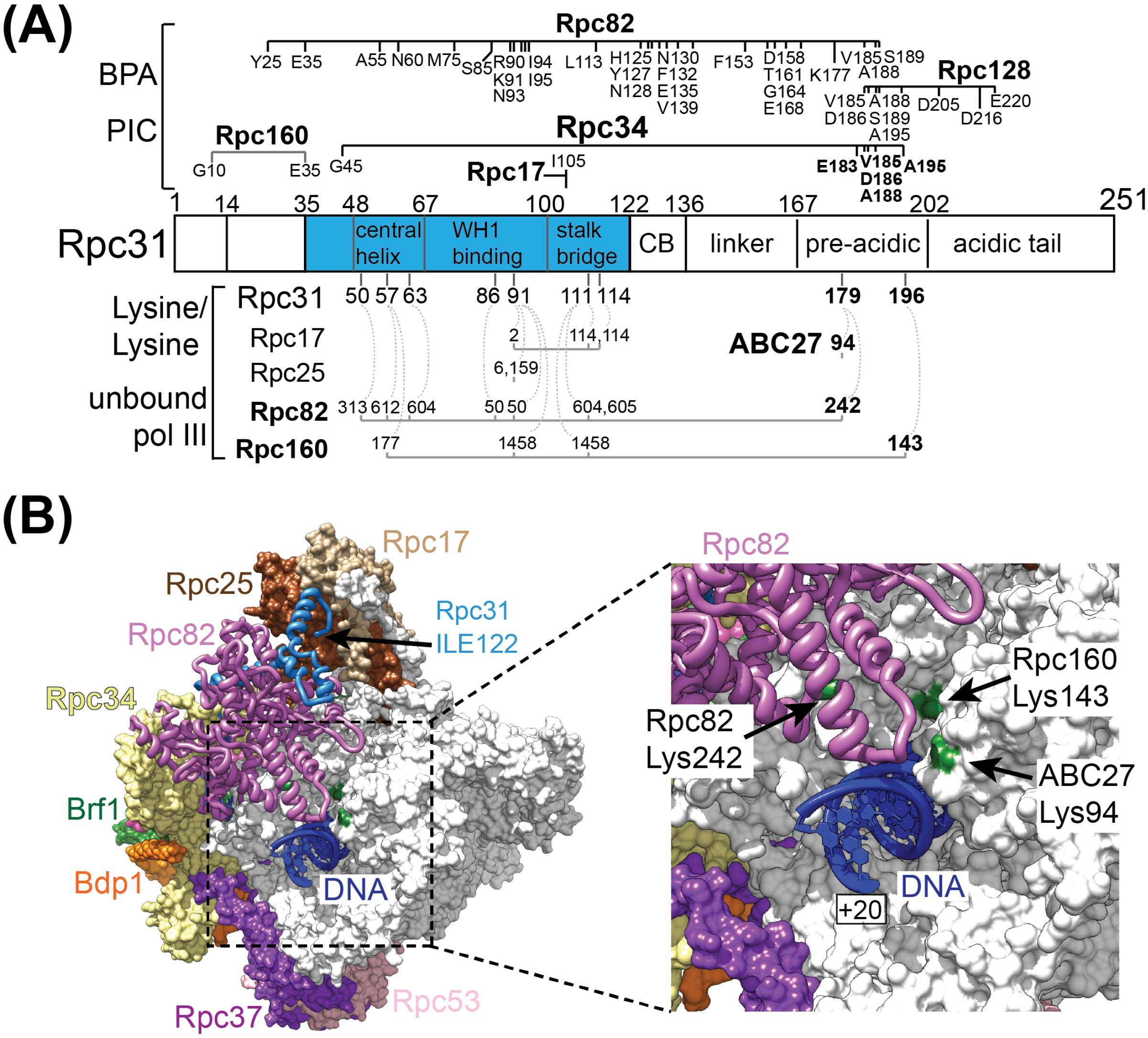

Supplementary Figure S6. (A) Summary of Rpc31 protein interaction mapping by BPA and lysine-specific crosslinking. Positions of BPA cross-links are shown above the schematic of Rpc31. Below the schematic, lysine residues of Rpc31 involved in lysine-specific crosslinking with Rpc17, Rpc25, Rpc82, Rpc160, and ABC27 are listed. Cross-linked lysines in respective pol III subunits are indicated. Dotted lines connect the cross-linked lysine pairs. As indicated, the BPA and lysine-specific cross-linking studies were respectively conducted in the isolated PICs and the purified unbound (apo) pol III. **(B)** Positions of lysines involved in cross-linking with the pre-acidic region of Rpc31. The structural model of pol III PIC is illustrated as in Fig. 1A, except that Rpc82 is displayed with the backbone trace model in purple. As illustrated in the enlarged view on the right, the positions of Lys143 of Rpc160, Lys94 of ABC27, and Lys242 of Rpc82 (all colored in green) are located adjacent to the region of the active site tunnel for the incoming duplex DNA (the DNA entry surface).

Supplementary Figure S7

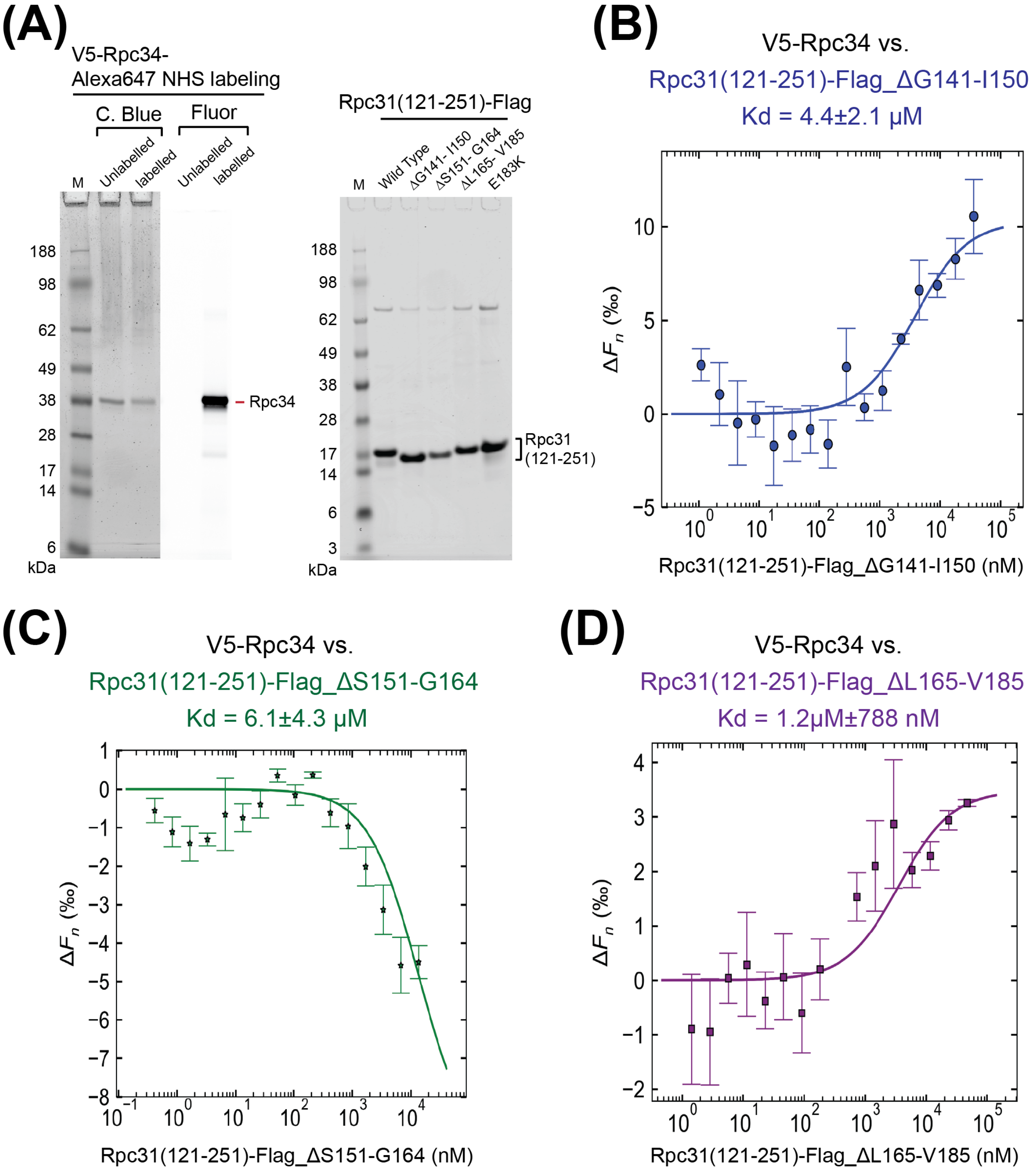

Supplemental Figure S7. Determination of the dissociation constant between Rpc31(aa. 121-251) peptide and Rpc34. (A) SDS-PAGE and Coomassie staining of purified polypeptides. The gels on the left are purified unlabeled Rpc34 and Alexa 647-labeled Rpc34 as indicated. Purified wild-type and mutated Rpc31(aa.121-251) peptides are shown in the gel on the right. **(B)-(D)** MST binding curves for the interaction between Rpc31(aa. 121-251) and Rpc34. Conditions for the MST experiment are as described in Figure 7. Deleted amino acids in the Rpc31(aa.121-251) peptide are as indicated.

Supplementary Table S1

Summary of BPA substitutions in Rpc31 and cross-linked polypeptides.

Position	Cross-linking	Cross-linked protein	Cα to Cα distance from Rpc31-BPA to the cross-linked protein[1]
R5	NO	-	
G10	YES	Rpc160	
Y15	NO	-	
P20	NO	-	
Y25	YES	Rpc82	
E35	YES >1	Rpc160 and Rpc82	14.4 Å (Rpc160) and 8.6 Å (Rpc82)
P40	NO	-	
G45	YES	Rpc34	8 Å (Rpc34)
A55	YES	Rpc82	6.3 Å (Rpc82)
N60	YES	Rpc82	9.1 Å (Rpc82)
V65	NO	-	
F70	NO	-	
M75	YES	Rpc82	8.6 Å (Rpc82)
D80	NO	-	
S85	YES >1	Rpc82 and ND[3]	9 Å (Rpc82)
R90	YES	Rpc82	6.5 Å (Rpc82)
K91	YES	Rpc82	6.9 Å (Rpc82)
N93	YES	Rpc82	9.3 Å (Rpc82)
I94	YES	Rpc82	7.2 Å (Rpc82)
I95	YES	Rpc82	10.6 Å (Rpc82)
I105	YES	Rpc17	22.9 Å (Rpc17)[2]
L113	YES	Rpc82	19.7 Å (Rpc82)[2]
I120	NO	-	
I122	NO	-	
D124	NO	-	
H125	YES	Rpc82	
Y127	YES	Rpc82	
N128	YES	Rpc82	
N130	YES	Rpc82	
L131	YES	ND	
F132	YES	Rpc82	
N134	NO	-	
E135	YES >1	Rpc82 and ND	
Y137	NO	-	
V139	YES	Rpc82	
M140	NO	-	
N143	NO	-	
K145	NO	-	
L148	NO	-	
I150	NO	-	
F153	YES	Rpc82	

D158	YES	Rpc82	
T161	YES	Rpc82	
G164	YES	Rpc82	
L165	NO	-	
E168	YES >1	Rpc82 and ND	
S173	NO	-	
L175	NO	-	
K177	YES >1	Rpc82 and ND	
E180	NO	-	
E183	YES	Rpc34	
V185	YES >1	Rpc34, Rpc82, and Rpc128	
D186	YES >1	Rpc34 and Rpc128	
A188	YES >1	Rpc34, Rpc82, and Rpc128	
S189	YES >1	Rpc82 and Rpc128	
A195	YES >1	Rpc34 and Rpc128	
D205	YES	Rpc128	
D210	NO	-	
D216	YES	Rpc128	
E220	YES	Rpc128	
Y225	NO	-	
N226	NO	-	
K229	NO	-	
Y230	NO	-	
F231	NO	-	
D235	NO	-	
G240	NO	-	
P245	NO	-	

1. The Cα to Cα distance is derived from the cryo-EM structure of an initiating pol III complex, PDB code 6eu0 (5).
2. The cross-linked polypeptide is located outside the cross-linkable distance range of ~10 Å (Cα to Cα) based on the cryo-EM structure of an initiating pol III, PDB code 6eu0.
3. “ND” indicates the cross-linked polypeptide was not determined in the study.

References

1. Girbig, M., Misiaszek, A.D., Vorländer, M.K., Lafita, A., Grötsch, H., Baudin, F., Bateman, A. and Müller, C.W. (2021) Cryo-EM structures of human RNA polymerase III in its unbound and transcribing states. *Nat Struct Mol Biol*, **28**, 210-219.
2. Wang, Q., Li, S., Wan, F., Xu, Y., Wu, Z., Cao, M., Lan, P., Lei, M. and Wu, J. (2021) Structural insights into transcriptional regulation of human RNA polymerase III. *Nat Struct Mol Biol*, **28**, 220-227.
3. Thompson, J.D., Higgins, D.G. and Gibson, T.J. (1994) CLUSTAL W: improving the sensitivity of progressive multiple sequence alignment through sequence weighting, position-specific gap penalties and weight matrix choice. *Nucleic acids research*, **22**, 4673-4680.
4. Gouet, P., Robert, X. and Courcelle, E. (2003) ESPript/ENDscript: extracting and rendering sequence and 3D information from atomic structures of proteins. *Nucleic acids research*, **31**, 3320-3323.
5. Abascal-Palacios, G., Ramsay, E.P., Beuron, F., Morris, E. and Vannini, A. (2018) Structural basis of RNA polymerase III transcription initiation. *Nature*, **553**, 301-306.
